# Regulatory modules of human thermogenic adipocytes: functional genomics of large cohort and Meta-analysis derived marker-genes

**DOI:** 10.1186/s12864-021-08126-8

**Published:** 2021-12-11

**Authors:** Beáta B. Tóth, Zoltán Barta, Ákos Barnabás Barta, László Fésüs

**Affiliations:** 1 Department of Biochemistry and Molecular Biology, Faculty of Medicine, University of Debrecen, Egyetem Tér 1, Debrecen, H-4032 Hungary; 2 MTA-DE Behavioural Ecology Research Group, Department of Evolutionary Zoology and Human Biology, University of Debrecen, Egyetem tér 1, Debrecen, H-4032 Hungary; 3 Vienna University of Economics and Business (WU), Welthandelspl. 1, 1020 Wien, Austria

**Keywords:** Adipocytes, browning and thermogenesis, Protein interaction networks, gene expression regulation, RNA-seq data, Transcriptional factors, HIF1A, UCP1 promoter, AdipoNET

## Abstract

**Background:**

Recently, ProFAT and BATLAS studies identified brown and white adipocytes marker genes based on analysis of large databases. They offered scores to determine the thermogenic status of adipocytes using the gene-expression data of these markers. In this work, we investigated the functional context of these genes.

**Results:**

Gene Set Enrichment Analyses (KEGG, Reactome) of the BATLAS and ProFAT marker-genes identified pathways deterministic in the formation of brown and white adipocytes. The collection of the annotated proteins of the defined pathways resulted in expanded white and brown characteristic protein-sets, which theoretically contain all functional proteins that could be involved in the formation of adipocytes. Based on our previously obtained RNA-seq data, we visualized the expression profile of these proteins coding genes and found patterns consistent with the two adipocyte phenotypes. The trajectory of the regulatory processes could be outlined by the transcriptional profile of progenitor and differentiated adipocytes, highlighting the importance of suppression processes in browning. Protein interaction network-based functional genomics by STRING, Cytoscape and R-Igraph platforms revealed that different biological processes shape the brown and white adipocytes and highlighted key regulatory elements and modules including GAPDH-CS, DECR1, SOD2, IL6, HRAS, MTOR, INS-AKT, ERBB2 and 4-NFKB, and SLIT-ROBO-MAPK. To assess the potential role of a particular protein in shaping adipocytes, we assigned interaction network location-based scores (betweenness centrality, number of bridges) to them and created a freely accessible platform, the AdipoNET (https//adiponet.com), to conveniently use these data. The Eukaryote Promoter Database predicted the response elements in the *UCP1* promoter for the identified, potentially important transcription factors (HIF1A, MYC, REL, PPARG, TP53, AR, RUNX, and FoxO1).

**Conclusion:**

Our integrative approach-based results allowed us to investigate potential regulatory elements of thermogenesis in adipose tissue. The analyses revealed that some unique biological processes form the brown and white adipocyte phenotypes, which presumes the existence of the transitional states. The data also suggests that the two phenotypes are not mutually exclusive, and differentiation of thermogenic adipocyte requires induction of browning as well as repressions of whitening. The recognition of these simultaneous actions and the identified regulatory modules can open new direction in obesity research.

**Supplementary Information:**

The online version contains supplementary material available at 10.1186/s12864-021-08126-8.

## Background

Directing our bodies to burn excess energy by up-regulating high-energy releasing biochemical reactions at the cellular level is a major topic of metabolic research, given the rise of obesity and related pathologies worldwide. One promising therapeutic solution to combat obesity is weight loss induced by thermogenesis in adipose tissue [1, 2]. The need for a pharmacological treatment is therefore pressing. A growing number of newly identified genes and proteins have been shown to play a role in thermogenic processes in adipocytes; however, our knowledge about how to safely and effectively activate the heat production of the fat cells is still incomplete.

The key molecular marker of thermogenicity (browning) in adipose tissue is the expression of Uncoupling Protein 1 (UCP1), which dissipates the proton gradient in the mitochondrial inner membrane and generates heat [3, 4]. Further studies have discovered additional markers and regulatory elements [5, 6], which vary by tissue localization [7–9] and organism type [10, 11]. Moreover, with new approaches, such as single-nucleotide transcriptomic studies, the variability has expanded into a new dimension and explores intra-tissue heterogeneity [12–16]. Furthermore, the knowledge of the regulatory role of non-coding gene regions and RNAs, such as miRNAs and lnc RNAs has also increased [17, 18] and calls for further research. In addition, based on genome-wide association studies, a single nucleotide polymorphism (SNP), such as at the locus of the Fat mass and obesity-associated (*FTO*) gene intronic region rs1421085, can critically contribute and modify the development of thermogenic phenotype [19, 20]. Claussnitzer [19] demonstrated that the presence of the homozygous C/C obesity-risk alleles in this *FTO* locus results in the expression of nearby genes *IRX3* and *IRX5*, thus generating less thermogenic adipocyte phenotype. By contrast, in T/T non-obesity risk (higher thermogenic potential) genotype individuals, the ARID5B repressor binds to this DNA site and thus blocks the expression of *IRX3* and *IRX5,* allowing the formation of the browning adipocytes. These data show that the characterization of the human brown adipocyte phenotype is still a challenge and emphasizes the high complexity of the thermogenic processes in adipocytes.

Recently, transcriptome-based studies of large cohort datasets (BATLAS [21];) or meta-analyses of available experimental results (ProFAT [22];) found a distinct molecular signature for the brown and white adipocyte phenotypes and identified some basic marker-genes for phenotype prediction. They offered scores to determine the thermogenic status of adipocytes using gene-expression data of these markers. In exploring bulk transcriptomic data, the variability in results from different experimental conditions can be overcome, providing the general picture of the investigated biological process. BATLAS and ProFAT marker-gene based scores evaluate the thermogenic status of tissue or adipose cell using the expression patterns of these marker-genes (BATLAS 98 brown and 21 white genes, Additional file 6, Supplementary Table 1 [21].; ProFAT 53 brown and 6 white genes; Additional file 7, Supplementary Table 2 [22].) and, therefore, provide comprehensive and standardized approaches. In the present study, we intended to investigate the functional context of the two marker-gene sets.

Interestingly, the two sets of genes have little overlap: only 17 common genes can be found (Additional file 10, Supplementary Fig. 1). However, by using other approaches to examine these genes, we found more agreement. Both gene-sets mainly encode proteins function in the mitochondria (considering MitoCarta 2.0 database [23];); in fact, 85 and 86% of the browning marker-genes encode mitochondrial proteins in BATLAS and ProFAT, respectively, while none of the white markers do. By performing Gene Set Enrichment Analyses (GSEA; we used KEGG and REACTOME pathway analyzers which are integrated into the STRING computer tool (https://string-db.org [24];), we found a clear consistency in the enriched pathways of the two brown marker-gene sets, which suggests that these pathways may be critical and deterministic in generating the thermogenic phenotype. The collected annotated proteins of the defined pathways provided a new expanded white and brown characteristic protein-set and their coding gene-set. It theoretically contains all functional proteins that could be involved in the formation of the adipocyte phenotype. Therefore, we further analyzed these proteins and their coding genes to get generally applicable conclusions.

To visualize the relative expression profile of these proteins coding genes (expanded white and brown gene-sets) in adipocytes, we used transcriptional data from our recent study [25] on ex vivo differentiated primary human subcutaneous (SC) and deep neck (DN) brown adipocytes, with or without *FTO* rs1421085 intronic SNP (obesity-risk allele). In the previous study, we found that irrespective of the tissue locality and the applied differentiation protocol (thermogenic induction during differentiation: brown protocol; No thermogenic induction: white protocol; Methods), the molecular signature of the *FTO* normal (T/T) genotype adipocytes suggests higher thermognicity than the *FTO* obesity-risk genotype (C/C) adipocytes. Therefore, in this study, we generated heatmaps and compared the gene-expression patterns based on this approach. We found that the white and brown expanded gene-sets expression pattern consistent with the adipocyte phenotype. Moreover, the trajectory of the regulatory processes is outlined by the transcriptional profile of pre-and differentiated adipocytes and highlights the importance of the suppression processes in brown adipocytes. The protein interaction network-based functional genomics indicate that different biological processes shape the brown and white adipocytes and point to the key regulatory elements and modules. The core module of their interactome identifies the unique signaling pathways and transcriptional regulatory elements that may determine the adipocyte phenotype and provides new targets in the pharmacological treatment of obesity. To assess the potential role of a particular protein or gene in shaping the adipocyte phenotype, we assigned interaction network location-based scores (betweenness centrality, number of bridges) to them and created a freely accessible platform, the AdipoNET (https//adiponet.com), for the convenient use of these data.

## Construction and content

### General methods

#### Generation of the expanded protein/gene set

**Fig. 1:**
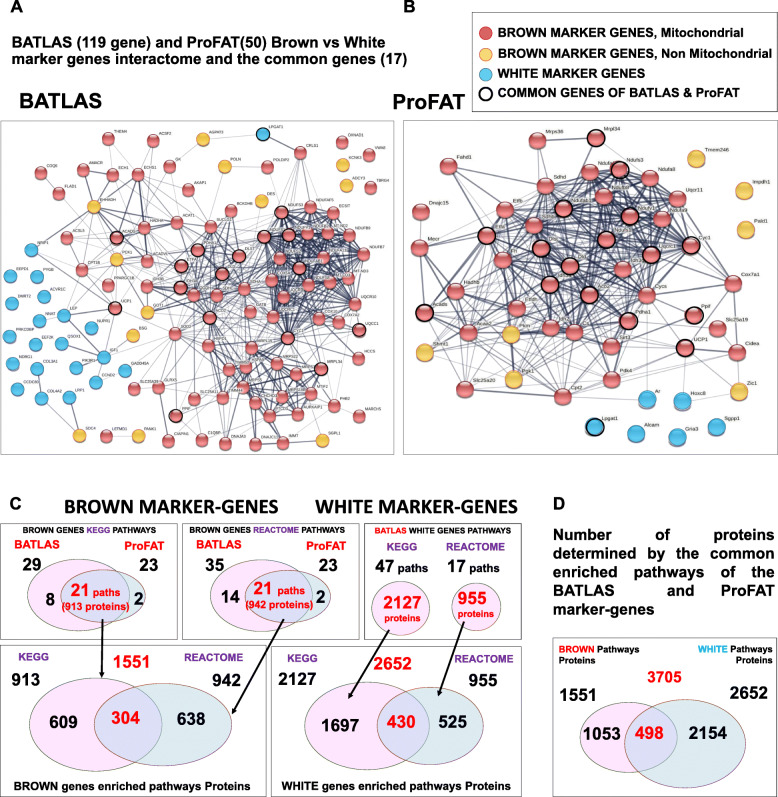
Interactome and Pathway analyses of the BATLAS and the ProFAT marker-genes. Interactome map of (**A**) BATLAS and (**B**) ProFAT brown and white marker-genes; interaction score confidence value: 0.4 (**C**) Venn diagrams show the pipeline of the expanded gene-set generation; the number of the KEGG and REACTOME pathways enriched by the marker-genes from BATLAS and ProFAT database and the number of the white and brown pathway associated proteins. (**D**) Venn diagram summarizes the number of proteins determined by the enriched pathways of the BATLAS and ProFAT marker-genes

BATLAS and ProFAT brown and white marker-genes (basic marker-genes) were subjected to Gene Set Enrichment Analysis (GSEA) separately. We explored the two marker-gene lists to identify enrichment of specific Functional Protein Sets annotated to certain pathways. Enriched pathway: annotated proteins/genes of the pathway are represented in the two gene-sets significantly higher than the random probability. The enriched pathways were identified by open source, the Kyoto Encyclopedia of Genes and Genomes (KEGG) and REACTOME pathway analyzer, which are integrated into the STRING computer tool (software) (https://string-db.org [24];). Based on the KEGG (https://www.genome.jp/kegg/pathway.html as of February 2020) [26] and REACTOME (http://www.reactome.org as of February 2020) [27] databases, we retrieved annotated protein sets for the identified enriched pathways. These databases characterize each pathway only with the participating proteins and do not contain regulatory, non-coding elements, therefore, the role of the noncoding RNAs cannot be directly measured by analyzing this database. We worked with the annotated proteins of pathways enriched for marker genes, and based on this, generated the expanded protein and the corresponding gene sets. To generate the expanded brown protein/gene set, only proteins belonging to those pathways that were enriched for both BATLAS and ProFAT were collected (Fig. 1C, D, Table 1) in the case of the basic brown marker-genes. In the case of the basic white marker-genes, only the BATLAS genes enriched pathways were used, because, in the case of ProFAT, the 6 white genes did not define any enriched pathway (Fig. 1C, D). Pathways that are too general and have a large number of proteins (more than 600) were excluded from the analysis; thus Metabolism (1250 KEGG proteins, 2032 REACTOME proteins) and Metabolism of Lipid (721 REACTOME proteins) among brown proteins and Signal Transduction (2605 REACTOME proteins) and Developmental Biology (1023 REACTOME proteins) pathways for white genes were excluded from the expanded gene sets (Additional files 1, 2, 3 and 4).

**Table 1:** Common KEGG and REACTOME enriched pathways in BATLAS and ProFAT brown Marker-genes. Table 1 shows the 21 KEGG and 21 REACTOME pathways that were enriched in both BATLAS and ProFAT brown-markers

Common KEGG enriched pathways in ProFAT and BATLAS Brown Marker-genes	Common REACTOM enriched pathways in BATLAS and ProFAT Brown Marker-genes
**Metabolic pathways**	**The citric acid (TCA) cycle and respiratory electron transport**
**Citrate cycle (TCA cycle)**	**Metabolism**
**Carbon metabolism**	**Pyruvate metabolism and Citric Acid (TCA) cycle**
**Thermogenesis**	**Respiratory electron transport, ATP synthesis by chemiosmotic coupling, and heat production by uncoupling proteins.**
**Parkinson’s disease**	**Citric acid cycle (TCA cycle)**
**Huntington’s disease**	**Respiratory electron transport**
**Oxidative phosphorylation**	**Complex I biogenesis**
**Non-alcoholic fatty liver disease (NAFLD)**	**Mitochondrial Fatty Acid Beta-Oxidation**
**Alzheimer’s disease**	**Fatty acid metabolism**
**Biosynthesis of amino acids**	**Glyoxylate metabolism and glycine degradation**
**2-Oxocarboxylic acid metabolism**	**mitochondrial fatty acid beta-oxidation of saturated fatty acids**
**Fatty acid metabolism**	**Regulation of pyruvate dehydrogenase (PDH) complex**
**Retrograde endocannabinoid signaling**	**Beta oxidation of hexanoyl-CoA to butanoyl-CoA**
**Pyruvate metabolism**	**Beta oxidation of decanoyl-CoA to octanoyl-CoA-CoA**
**Fatty acid degradation**	**Signaling by Retinoic Acid**
**Valine, leucine and isoleucine degradation**	**Metabolism of lipids**
**Fatty acid elongation**	**Mitochondrial protein import**
**Glyoxylate and dicarboxylate metabolism**	**Lysine catabolism**
**Glycolysis / Gluconeogenesis**	**Metabolism of amino acids and derivatives**
**Cardiac muscle contraction**	**Branched-chain amino acid catabolism**
**PPAR signaling pathway**	**Transcriptional activation of mitochondrial biogenesis**

#### Origin of the transcriptomic data

Ethics statement for the obtained samples, isolation, and differentiation of human adipose stromal cells (hASCs), as well as RNA and DNA isolation, genotyping, RNA-sequencing is already described in Tóth et al. [25]. The hASCs were isolated from paired DN (Deep Neck) and SC (Subcutaneous Neck) adipose tissue samples of nine donors, three of each *FTO* rs1421085 genotype: T/T-risk-free, T/C-heterozygous, and C/C-obesity-risk. After cultivation, both brown and white differentiations were induced by hormonal cocktails in serum and additive-free DMEM-F12-HAM medium.

#### Heatmap visualization of the gene-expression data

Hierarchical cluster analyses and heat map visualization of the relative gene-expression data were performed in the Morpheus web tool (https://software.broadinstitute.org/morpheus/) using Pearson correlation of rows and complete linkage based on calculated z-score of DESeq normalized and filtered data (low-expressed and outlier) after log_2_ transformation [25]. Z-score is calculated as (X - m)/s, where X is the value of the element, m is the mean, and s is the SD. Heatmaps show samples 1–3 with *FTO* T/T-risk-free allele (donor 1–3) and samples 7–9 with C/C-obesity-risk alleles (donor 7–9); we left out the *FTO* T/C heterozygous samples (donor 4–6), which are shown for some case in the paper Tóth et al. [25].

#### Interactome analyses

We used the computational tool STRING (https://string-db.org [24];) to generate protein-based interactomes, where nodes represent a protein and edges represent protein-protein-based interactions. STRING and Cytoscape applications were used for the visualization of interactomes. To explore the interaction of proteins encoded by the expanded brown and white gene/protein-sets, we used the default median interaction confidence score (0.4) as a threshold. The STRING database considers seven specific types of evidence for the interaction of two proteins that contribute to the overall interaction confidence score, and we used all of them. Further analyses of the STRING network data were performed in the Igraph package ([28]; https://igraph.org.) of the R interactive statistical environment [29] to compute the betweenness scores and the number of bridges of the interacting nodes. We calculated the betweenness centrality score to identify important proteins in the most central positions connecting different functional modules and the number of bridges to learn how many modules are connected by this protein. Interaction score confidence values (0.4) were used to visualize the network, as indicated in the figures. Unsupervised MCL clustering was used from the ClusterMaker2 application downloaded in Cytoscape software to explore sub-clusters of the generated interactome of the 3705 Brown and White Pathways proteins. Interaction score confidence value: 0.4. MCL parameters: Granularity parameter: 2; Array source: stringdb: score; Edge weight cutoff: 80; No restored inter-cluster edges after layout. Limitation: the STRING database uses protein-protein interaction data to generate the linkage network, which eliminates the exploration of the role of non-coding RNAs directly. However, when the position in the network predicts a significant role of a protein, the known factors that regulate it, such as miRNAs and lncRNAs may be traced back.

#### Isolation, cell culture, differentiation and treatments of hASCs

The hASCs were isolated from Subcutaneous (SC) adipose tissue. The volunteer, 48 year with a BMI of 19.7, underwent a planned cosmetic surgical treatment. At the time of the surgery, patients were without malignant tumor, diabetes, or abnormal thyroid hormone levels. hASCs were isolated and cultivated following the procedure in Tóth et al. [25]. The absence of mycoplasma was checked by polymerase chain reaction (PCR) analysis (PCR Mycoplasma Test Kit I/C, PromoCell cat# PK-CA91). The SC abdominal adipocytes from hASCs were isolated and grown till confluence state, then differentiated with white differentiation cocktail on 6 well plates at 37 °C in 5% CO_2_, with a white cocktail, according to previously described protocols [25]. Briefly, cells were grown in DMEM-F12-HAM (Sigma-Aldrich cat#D8437) medium supplemented with 10% FBS (Gibco cat#10270106); 10% EGM2 (Lonza #CC3162), 1% Biotin (Sigma-Aldrich cat#B4639), 1% Pantothenic acid (Sigma-Aldrich cat#P5155), 1% Streptomycin-Penicillin (Sigma-Aldrich cat#P4333): white differentiations were induced at the first 4 days by hormonal cocktails in serum and additive-free DMEM-F12-HAM (Sigma-Aldrich cat#D8437) medium that contain biotin, pantothenic acid, apo-transferrin 10μg/ml (Sigma-Aldrich cat#T2252), insulin 20 nM (Sigma-Aldrich cat#I9278), T3 3nM (Sigma-Aldrich cat#T6397), dexamethasone 25 nM (Sigma-Aldrich cat#D1756), hydrocortisone (Sigma-Aldrich cat#H0888) rosiglitazone 2 uM (Cayman Chemicals cat#71740), and IBMX 250 uM (Sigma-Aldrich cat#I5879). Later, for 10 days, rosiglitazone, dexamethasone, and IBMX were omitted from the media. To compose the brown media for thermogenic induction serum and additive-free DMEM-F12-HAM (Sigma-Aldrich cat#D8437) medium that contains biotin, pantothenic acid, apo-transferrin 10μg/ml (Sigma-Aldrich cat#T2252), insulin 850 nM (Sigma-Aldrich cat#I9278), T3 3nM (Sigma-Aldrich cat#T6397), and rosiglitazone 500 nM (Cayman Chemicals cat#71740) were present in the cocktail. Insulin was present at 42.5x higher concentration than in the white differentiation cocktail.

#### Thermogenic activation and hypoxic experiment

Differentiated adipocytes on 6 well plates were used for the experiment. Two treatments were performed in parallel (3 wells/treatment): Thermogenic cocktail (Brown media supplemented with 500 μM cAMP analog (dibutyryl-cAMP; Sigma-Aldrich cat#D0627) [9]) was applied for 16 h to induce thermogenesis or hypoxic condition (1% O_2_) was maintained for 16 h in a hypoxic chamber. The control group was the untreated differentiated adipocytes. The hypoxic gas-mix contains 1% O_2_, 5% CO_2_, 94% N_2_.

#### Western blotting

The cellular lysate was obtained using 2X denaturing buffer (100 ml: 25 ml TRIS pH 6.8; 45 ml MilliQ Water; 20 ml glycerol; 10 ml MEA; 4 g SDS, Bromophenol Blue) diluted to 1:1 with urea (8 M). Briefly, 20 μl protein from cell culture lysates was separated using 10% acrylamide gel and transferred to PVDF Immobilon-P Transfer Membrane (Merck-Millipore, Darmstadt, Germany); blocked with 5% milk powder in TTBS (0.1% Tween 20 in TBS), and incubated overnight at 4 °C separately with UCP1 (1:750 dilution in 1% milk powder in TTBS; R&D Systems, Minneapolis, MN, USA, MAB6158), HIF1A (1:750 dilution in 1% milk powder in TTBS; BD cat#610958) and beta-Tubulin (1:10000 dilution in 1% milk powder in TTBS Sigma-Aldrich cat#F2168) antibodies. Following overnight incubation, blots were incubated with HRP-conjugated secondary anti-mouse antibody for 1 h (1:5000 dilutions in 1% milk powder in TTBS; 1:5000, Advansta, R-05071–500) and exposed to x-ray films using chemiluminescent detection method (ECL, Merck-Millipore, MA, USA).

#### Transcription factors binding sites in the UCP1 promoter

The eukaryotic promoter database (EPD; https://epd.epfl.ch) was used to determine whether the Transcription Factor (TF) has binding sites (response element) in the *UCP1* promoter region [30]. The EPD contains comprehensive organisms-specific transcription start site (TSS) collections automatically derived from next-generation sequencing (NGS) data and corresponding promoter region (− 5000 and + 100 Base pairs from TSS). We use the library of the TF Motifs (JASPAR CORE 2018 vertebrates [31];). The consensus sequence of the TFs was identified by the JASPAR (http://jaspar.genereg.net/) database, which is an open-access database of curated, non-redundant TF-binding profiles [31].

Chip-Atlas (http://dbarchive.biosciencedbc.jp [32];), ChIPSummitDB database (University of Debrecen; http://summit.med.unideb.hu [33];), ENCODE (https://genome-euro.ucsc.edu), and EnhancerDB (http://lcbb.swjtu.edu.cn/EnhancerDB) were used to investigate the experimental data about HIF1A and other TFs binding to the *UCP1* and *UCP2* promoters. These online tools collect, organize and visualize the results of the published chip-seq. experiments. The chromosome region is given according to the hg19 (GRCh37) human reference genome in the case of ChIPSummitDB; otherwise, the GRCh38/hg38 genome assembly was used.

#### Comparative genomic analyses

For comparative genomic analysis, we used the UCSC genome browser (University of California Genomics Institute, Santa Cruz, https://genome-euro.ucsc.edu) and tracked the close vicinity of the HIF1A response element in the *UCP1* promoter. The tracking shows multiple alignments of 100 vertebrate species and measurements of evolutionary conservation.

## Utility and discussion

### GSEA of BATLAS and ProFAT marker-genes reveals characteristic biological pathways for thermogenic and white adipocytes

First, to find which biological processes and pathways were associated with the basic marker-genes, we performed GSEA separately for the brown and white markers of BATLAS and ProFAT (Additional file 8, Supplementary Table 3. A, B, C, D; note that the six ProFAT white genes did not define any enriched pathway; Methods). Brown marker-genes showed high consistency in the enriched pathways: of the 23 and 23 significantly enriched KEGG ([26]; see also in Methods) and REACTOME ([27]; Methods) pathways defined by the ProFAT brown marker-genes, respectively, almost all were also enriched by analysing the BATLAS brown marker gene set (21 for KEGG and 21 for REACTOME) (Table 1, Additional file 8, Supplementary Table 3. A, B, red color). This suggests that these commonly enriched pathways are critical in generating the thermogenic phenotype given that the two, large database and meta-analyses studies (BATLAS and ProFAT), that used different databases and selecting algorithms, identified mainly dissimilar genes from almost the same pathways. Importantly, enriched pathways defined by the BATLAS and ProFAT brown marker-genes were also found to be significantly enriched based on the preferentially expressed genes in our previous study [25] of the non-obese *FTO* genotype brown adipocytes (Additional file 8, Supplementary Table 3 A, B, blue color), further emphasizing their role in shaping the thermogenic phenotype. The pathways defined by the brown marker-gene sets (BATLAS, ProFAT) were almost different from those defined by the BATLAS white marker-genes (47 KEGG and 17 REACTOME pathways; Additional file 8, Supplementary Table 3 C, D). Only the Non-alcoholic fatty liver disease (NAFLD) KEGG pathway was identified by both gene sets. Furthermore, in both cases, the STRING [24] interaction network of the proteins coded by the marker-genes (Fig. 1 A,B) show that while there is a high degree of interaction among the brown marker proteins, there is hardly any among the white markers and brown and white markers do not form an integrated network but appear side by side. Therefore, these findings suggest that fundamentally different processes underlie the development of the two adipocyte phenotypes, and those entire pathways may better characterize the phenotype than marker-genes.

### Annotated proteins from the enriched pathways provides expanded protein and gene sets to explore the regulation of adipocyte thermogenicity

Considering the high consensus of the identified pathways of BATLAS, ProFAT, and the preferentially expressed genes of the FTO non-obese genotype brown adipocytes, we can reasonably assume that all proteins/genes belonging to the commonly enriched brown and white pathways may play a role in the formation of the adipocyte phenotype. This allows us to investigate the potential regulatory elements of thermogenesis in adipose tissue, so that the origin of the gene/protein is not limited to adipocytes, all significant but otherwise condition-dependent processes could be involved, and markers that are not regulated primarily at the gene expression level can also be recognized. These advantages prompted us to collect all the proteins associated with the BATLAS and ProFAT brown and white pathways and their coding genes to generate expanded brown and white protein/gene-sets (Additional files 1, 2, 3 and 4). Further, we continued the systematic analysis with these expanded protein and gene sets to better explore the potential regulatory processes in thermogenesis and understand the special relationship between genes forming the brown (thermogenic) and white (non-thermogenic) adipocyte phenotype. The implemented pipeline for generating and analyzing the expanded brown and white protein/gene-set is summarized in Figs. 1C, D, and Additional file 10, Supplementary Fig. 2 A, B, C (see also in the methodology section). Altogether 3705 proteins, 1551 brown, annotated to 44 pathways (brown pathway proteins/genes) and 2652 white, annotated to 64 pathways (white pathway proteins/genes) were identified by the enriched pathways derived from BATLAS and ProFAT marker-genes when KEGG and REACTOME analyses results were combined (Fig. 1C, D; Additional files 1, 2, 3 and 4). The KEGG and REACTOME pathway analyzers use different approaches, as only a minority of the proteins/genes were identified by both (304 in the case of the brown and 430 in the case of the white marker-genes). Although the basic brown marker-gene sets defined pathways differ from those of the basic white marker-genes, there were overlaps between the annotated proteins of the pathways (Fig. 1D). These 498 common proteins may confer and interface between the two phenotypes balancing the regulation of the thermogenic capacity of the adipocytes; hence, we named them linker proteins and genes. Not all the 3705 identified proteins/genes (expanded protein/gene set) are expressed in adipocytes based on our study with neck adipocytes, but they can be involved in the higher-level regulatory processes of thermogenesis. Therefore, we worked with all the proteins/genes in the following network analyses; however, the heatmaps of the gene-expression profile only depict those genes expressed in adipocytes from our previous study [25] to increase the clarity of our visualizations.

### The expression profile of the expanded gene-sets is consistent with adipocyte phenotypes

**Fig. 2:**
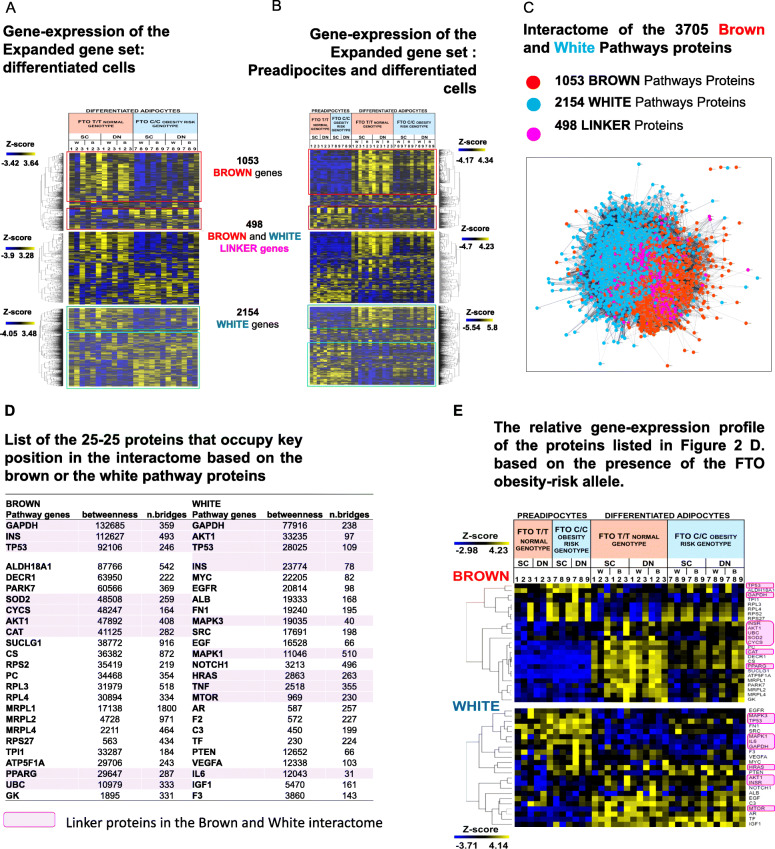
Gene-expression profile and interactome analyses of the Expanded gene set from the Brown and White pathway genes. (**A**) Heatmaps show separately the relative gene-expression profile of the genes enriched in the Brown, the White, and both the Brown and White pathways (Linker genes) in differentiated adipocytes and (**B**) in preadipocytes and differentiated adipocytes based on the presence of FTO obesity-risk alleles; we used samples with *FTO* T/T-risk-free (donor 1–3) and C/C-obesity-risk alleles (donor 7–9) and left out the *FTO* T/C heterozygous samples (donor 4–6), which are shown for some cases in the paper Tóth et al., 2020. B: Brown differentiation; W: White differentiation; SC: Subcutaneous; DN: Deep-neck (**C**) Interactome of the 3705 Brown and White Pathways proteins; Interaction score confidence value: 0.4. (**D**) List of 25–25 proteins that occupy a pivotal position in the interactome based on the betweenness centrality score (betweenness) and the number of bridges (n.bridges) from network analyses of the Brown or the White pathway proteins. Magenta Highlights the Linker-proteins that occupy a pivotal position in the interactome based on the Brown or White pathway proteins. (**E**) The relative gene-expression profile of the 25–25 proteins with the highest betweenness centrality score and number of bridges from the Brown and White pathway proteins based on the presence of the FTO obesity-risk allele. Magenta highlights the linker proteins

To explore how genes in the expanded sets are involved in the formation of a particular adipocyte phenotype, their relative expression profiles in mature adipocytes are shown on three separate heatmaps, namely Brown (1053), Linker (498), and White (2154) genes, as they manifest according to the presence/absence of the *FTO* obesity-risk allele in the above-mentioned study using human neck adipocytes [25] (Fig. 2A). The majority of the genes belonging to the Brown pathways have relatively higher expression in the more thermogenic samples: 540 genes, e.g., *DIO2, FABP3–4, PDK2–4, PC, CKMT1A, ELOVL3, DHRS9, LPL,* and *PLIN1,2,4,5* (light red box, note: exemplified genes here and below are show significantly differential expression between the *FTO* normal and obese genotype samples, however, not all genes belongs to the cluster reach differential expression at the *p* < 0.05 significant levels, but the heatmap suggests a trend). On the other hand, most of the White genes are more highly expressed in the less-thermogenic samples: 1211 genes, e.g., *IGF2, HAPLN1, NOX4, COMP, IL11, VCAM1, ID1, LEFTY2,* and *ADRA2C* (light green box)). These genes may be directly and positively involved in the development and/or maintenance of the given phenotype. Also, a smaller portion exhibit an opposite expression profile, suggesting a negative regulatory role in the pathway shaping a given brown (dark-red box) or white (dark-green box) phenotype. Some genes also show no differential expression profile, which may be because the activities of the proteins encoded by these genes are not regulated at the gene expression level but via post-translational modification or splicing variation that allows a much quicker response to changing conditions. It is also possible that non-coding RNAs regulate the translational processes of these mRNAs, resulting in different protein amounts in brown and white adipocytes. Besides, a slight change in expression of these genes/proteins may be enough to trigger a significant effect in the signaling process due to their highly interactive nature. The Linker genes show higher (e.g., *HK2, PGC1A and B, LIPE, SOD2, CD36, LDHB, PDK1,* and *CPT1B*) or lower (e.g.*, GNAO1, GNG7, ADCY4, BDNF, SCD5, GNB3, CPT1A,* and *TGFB1*) expression in approximately equal proportions in samples of *FTO* normal genotypes, which may indicate an important regulatory role in both directions to switch on/off thermogenesis. This is also supported by the pathways enriched by these genes, among which thermogenesis is one of the most significant (Additional file 9, Supplementary Table 4). In summary, the expression profile of the expanded gene set largely follows that of the basic marker-genes (Additional file 10, Supplementary Fig. 3A, B). However, since it encompasses the complete biological processes, unlike the marker-gene sets, which typically only highlight positive regulatory participants, it identifies those involved in negative regulation and, therefore, provides a suitable basis for a more complex analysis of adipocyte thermogenicity.

### Trajectory of regulatory processes is outlined by the transcriptional profile of expanded gene-sets in progenitor and differentiated adipocytes

Gene expression patterns of adipocyte progenitor cells are usually not reported in studies addressing thermogenic potential, although it may be important to examine them to see not only the differences between matured adipocyte phenotypes but also the direction of the regulatory processes. Most of the genes of the expanded gene sets show differential expression between the two *FTO* genotype samples only after differentiation and do not differ at the progenitor stage. When preadipocytes gene-expression data are involved in the analyses, the relative expression difference between mature adipocytes *FTO* normal and obesity-risk samples was less pronounced due to the large difference between the preadipocytes and differentiated cells (Fig. 2A, B, red and green boxes. Note: similar expression profiles are presented by the BATLAS and ProFAT basic marker-genes when preadipocytes were also included in the analysis, Additional file 10, Supplementary Fig. 3 C.D). The main expression pattern of the differentiated cells remained the same. However, a comparison to the preadipocyte gene-expression reveals that many of the highly expressed Brown genes in the *FTO* normal brown adipocytes after differentiation are also expressed in matured obesity-risk samples, albeit to a lesser extent (Fig. 2B. red box). This indicates that the two genotype samples only differ at the induction level of these genes and are not actually suppressed in obesity-risk genotype samples, hence emphasizes the importance of proper controls. On the other hand, considering the clusters of Brown genes downregulated during differentiation (Fig. 2 A, B dark red box), these genes are indeed highly repressed in the normal *FTO* genotype brown adipocytes, especially when compared to preadipocytes and the difference between the two *FTO* genotypes reflects the extent of repression. This highlights the possible importance of the repressed genes, which so far, have received less attention in analyses of the thermogenic processes. The revealed 175 genes repressed during differentiation include *LTC4S, HACD4, LHPP*, *PRDM16*, *GPX3, GPX8*, *NNMT* (Additional file 5). The mostly suppressed White genes in *FTO* normal brown adipocyte samples in the neck area after differentiation are needed to form the white phenotype, and their presence could interfere with thermogenesis (Fig. 2B. green box). Upregulated white genes in *FTO* normal genotype brown adipocytes could be negative regulators of the white phenotype and not directly involved in the thermogenic processes (e.g., *TGFA, LPAR3, SLC2A4, MGST2, FGF1*, *LPIN1, DUSP6,* and *CSF2RA*); however, increases in their expression level may repress the white phenotype, opening the way for appropriate browning. In conclusion, a comparative analysis of differentiated adipocyte samples based on the relative gene expression profile can explore the trajectory of the regulatory processes when the status of preadipocytes is also included, suggesting that in addition to the upregulated genes, the downregulation of some genes or pathways could have a critical role in determining adipocyte cell fate.

### Network position based prediction of the key regulatory modules of adipocyte phenotypes

To determine which functional modules and regulatory elements play a central role in the process of adipocyte phenotype formation, we mapped the interaction network of the proteins obtained from the expanded white and brown protein sets, as all of them are potential participants. As expected, the number of interactions is high in both the brown or white proteins (Fig. 2C) since proteins of specific pathways make up the expanded brown and white protein and gene sets, and interaction levels are usually high among proteins belonging to a particular pathway. For that reason, the network shows a so-called “hairball” profile [34] in which the sub-clusters are not visible, while it clearly demonstates that  the brown (red) and white (blue) proteins are spectacularly separated, as was the case of the basic ProFAT and BATLAS marker-gene sets (Fig. 2 C; Fig. 1 A, B; note: similar network appeared, when interaction confidence score was 0.7, not shown). Unsupervised Markov Clustering Algorithm (MCL) was used to explore the sub-clusters (Cytoscape), and the majority (41 (73%) out of 56 clusters which contain more than 8 proteins) of them contain either BROWN and LINKER or WHITE and LINKER proteins, while only 27% have exclusive BROWN and WHITE proteins mixed (Additional file 10, Supplementary Fig. 4; Method). These further strengthen our conclusion that mainly different processes develop the brown and white adipocyte phenotypes, and we can presume the possible transitional states when these processes are present concomitantly with varying intensity. It also suggests that inhibiting the formation of one phenotype would not necessarily imply inducing the other and *vice versa*. Based on this, we propose that the healthy induction of the thermogenic adipocyte phenotype may require simultaneous repression of whitening and activation of browning.

It can be also observed that proteins common to both brown and white pathways are more likely to occur in the middle of the network landscape (Fig. 2C, magenta, 498 linker proteins), crossing the interface of the brown and white parts rather than coinciding with it. This may mean that some of these linker proteins do not only connect the two phenotypic processes but are also deeply embedded in the networks of one phenotype, and thus potentially more comprehensively affect the particular phenotype. To identify these proteins, we performed a separate interactome analysis of the brown and white pathway proteins when the linker proteins were involved in both: 25–25 proteins, with the highest Betweenness Centrality score and Bridges Number as shown (Fig. 2D), highlighting the linker proteins (magenta). These proteins possess the potential of contributing to form either the brown or white phenotype, but of these, linker proteins may be the ones that could connect and balance these two functions (magenta). Their expression profile (Fig. 2E; upper heatmap) suggests that among the brown pathway genes, the majority show higher expression in the *FTO* normal samples, indicating a possible positive regulatory role or the consequences of browning (e.g., *PPARG, SUCLG1, PARK7*). While *TP53*, *ALDH18A1, RPL3–4, RPS2,* and *27* are repressed in the *FTO* normal brown adipocytes, pointing to the importance of their downregulation in brown phenotype formation. On the other hand, the white genes *SRC*, *MAPK3,* and *FN1* show increased expression in the obesity-risk brown adipocytes (Fig. 2E; Lower Heatmap) and may be required for whitening and quieten the thermogenic processes. Interestingly, four of the linker proteins (GAPDH, INS/INSR, TP53, and AKT1) appear as key in maintaining the interactome integrity in both the expanded brown and white protein networks, as they appeared in both lists, therefore, emphasizing their pleiotropic function in the biological processes forming the thermogenic phenotype.

**Fig. 3:**
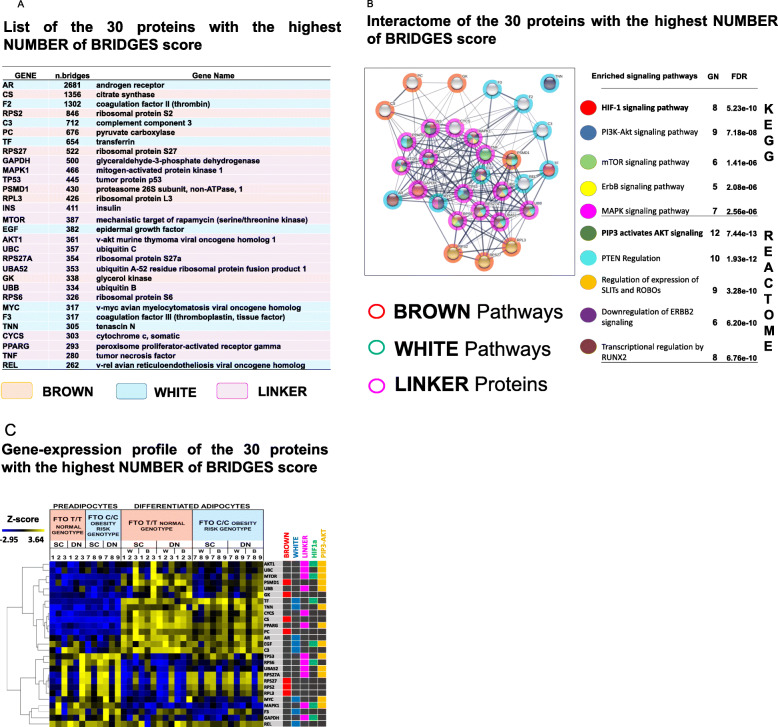
The list, Interactome analysis and relative gene-expression profile of the 30 proteins with the highest number of bridges in the network of the Expanded gene set. (**A**) The list of the 30 proteins with the highest NUMBER of BRIDGES score (core module-proteins) based on the interactome network analyses of the expanded protein-sets (3705 proteins) (**B**) Interactome of the 30 core-module proteins, the color of nodes marks the enriched pathways, and the color of rings shows the type of the phenotype the protein belongs; red: brown expanded protein-set, blue: white expanded protein-set, magenta: linker proteins; right panel lists the enriched signaling pathways, the number of proteins (NP) belonging to this pathway from the 30 core-module proteins and the significance level of the enrichment with False Discovery Rate value (FDR). (**C**) Gene-expression profile of the 30 core-module proteins, right panel marks genes that belong to expanded Brown gene set (red), expanded White gene set (blue), Linker genes (magenta), HIF1A pathway (green), and PIP3-AKT (yellow) pathway

In another approach, the expanded protein set derived from brown and white pathways (3705 genes) was considered as a whole, with the assumption that all of them could have a role (either positive or negative regulation) in determining the adipocyte phenotype. We identified 30 proteins that bridged the largest number of functional units (clusters) in the interaction network (Fig. 3A) and have potentially the greatest impact on others in the network; therefore, we considered them as the central pillars (core-module proteins) and further investigated their network relations, expression profile, and function. Most of them belong to both the brown and white pathways (linker genes/proteins; Fig. 3A. magenta) and are also closely related, as shown by their interactome (Fig. 3B). In addition, the linker proteins are aggregated in the middle, further supporting their possible central role in the formation of adipocyte phenotypes, as they can connect the distinct sub-processes that take place in differentiated adipocytes. According to KEGG and REACTOME pathway analyses, these 30 proteins belong to several pathways (the five most relevant ones are shown on the right side of the interactome figure (Fig. 3B)), therefore, highlighting their versatile role. The HIF1A and PIP3-AKT signalling pathways and PTEN regulation are the most significant modules, while the mTOR, MAPK, SLITs-ROBOs, ERBB2, and RUNX2 signaling pathways are also highly enriched by these proteins. The functions that can be related to them were explored by GSEA, and among them, the regulation of gene expression, the protein metabolic processes, the catalytic activity, and cell death emerged as the most significantly enriched biological pathways/processes. The Core-module proteins gene-expression profiles show that some of them are highly expressed in the *FTO* normal samples and may be positive effectors, while others show lower expression and can be involved as negative regulators in the process of differentiation to thermogenic adipocytes (Fig. 3C.). Thus, the interaction analysis of the expanded protein set emphasizes that different biological processes shape the two adipocyte phenotypes and that linker proteins could play a central role, especially some pleiotropic roles that can regulate both brown and white differentiation processes according to the particular condition. For the convenient use of the network position-based scores of the expanded protein/gene set, we developed the AdipoNET online platform (https//adiponet.com). This approach helps to explore/estimate the role of a given protein/gene in shaping the adipocyte phenotypes (BROWN, WHITE, or involved in both as a LINKER protein/gene).

### UCP1 promoter sequence analysis predicts the potential transcriptional regulatory elements such as HIF1A in adipocyte thermogenesis

**Fig. 4:**
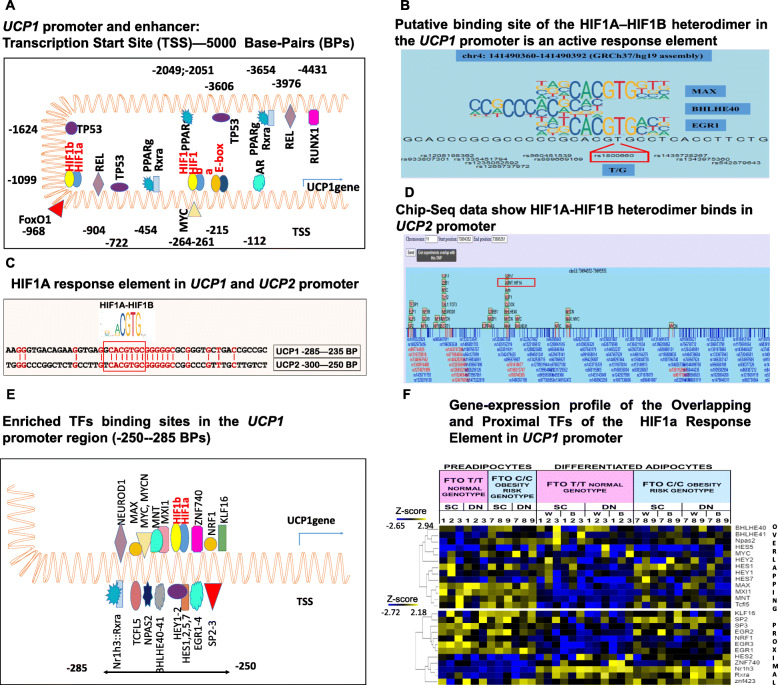
Promoter region of the *UCP1* and *UCP2* genes with enriched TF binding sequence and the gene-expression profile of these TFs. (**A**) The *UCP1* promoter and enhancer region from Transcription Start Site (TSS) to − 5000 Base-Pairs (BPs); TF binding site represent nucleotide position relative to TSS; the figure only shows TFs from the 30 core-module proteins (**B**) The chip-seq. data show TFs that bind to the identified HIFA response elements as well as in the immediate vicinity in the *UCP1* promoter and also show the SNPs coinciding with TFs binding sites. The figure was generated with the ChIPSummitDB online software. (**C**) Nucleotide sequence in the homologous promoter region (− 235--300 BPs) of the *UCP1* and *UCP2* genes (EPD database: *UCP1*, *UCP*2.2, Strand [−]), highlighting the similar region (red) and the HIF1A-HIF1B RE in a red box (EPD database). (**D**) The schematic figure shows TFs that bind in the promoter region (TSS - 1000 BPs) of the *UCP2* gene and also shows the SNPs coinciding with TFs binding sites. The figure was created with the ChIPSummitDB online software. The chromosome region is given according to the hg19 (GRCh37) human reference genome. Red-box shows HIF1A-ARNT among the binding TFs. (**E**) The *UCP1* promoter: -250--285 BPs show overlapping and proximal TFs binding sites with HIF1A-HIF1B response element. (**F**) Gene-expression profile of the TFs with Overlapping and Proximal binding site with HIF1A Response Element in *UCP1* promoter

Gene expression regulation was one of the significantly enriched pathways by the 30 core-module proteins, so we examined which Transcription Factors (TFs) appeared among them and in silico investigated whether they could directly regulate the gene expression of *UCP1*, the major thermogenic protein. MYC, REL, PPARG, TP53, and AR are among the 30 core-module proteins, so they can directly affect the expression of many other genes and thus may have a broad-spectrum effect (Fig. 3A). Furthermore, according to the Eukaryotic Promoter Database (EPD) (https://epd.epfl.ch) [30], they have their binding site sequence (Response Element; RE) in the *UCP1* promoter and/or enhancer region (− 5 MB-TSS; *p* < 0.0001; TP53 *p* < 0.001, Fig. 4A). We also investigated the three transcription factors that emerged in the significantly enriched signalling pathways, namely HIF1A, FoxO1, and RUNX. Intriguingly, they also have their binding sequence in the upstream DNA element of the *UCP1,* and the enrichment of the HIF1A RE has the highest probability (*p* < 0.00001; others *p* < 0.0001) (Fig. 4A). The role of HIF1A in obesity has been emphasized in hypoxic conditions but less studied in normoxic environments [30]. Although HIF1A expression is already reported after thermogenic induction in adipocytes even in UCP1 KO mice [35, 36], it is not fully clear whether a hypoxic condition is present in the cell or pseudo-hypoxic processes results in the stabilized HIF1A, and what function is associated with the stabilized HIF1A. Notwithstanding, it may suggests that the presence of HIF1A could not be only the consequence of thermogenic activation induced UCP1 expression triggered physiological changes, such as the reported increased ROS (Reactive Oxygen Species) production [37]. Furthermore, to investigate the functionality connected to the presence of HIF1A in thermogenic adipocytes, we checked the published chip-seq. data, however, we found no study HIF1A or HIF1B (ARNT) chip-seq in thermogenic adipocytes in ChIP-Atlas (http://dbarchive.biosciencedbc.jp [32];), ChIPSummitDB database (http://summit.med.unideb.hu [33];), ENCODE (https://genome-euro.ucsc.edu) or EnhancerDB (http://lcbb.swjtu.edu.cn/EnhancerDB), respectively. For other cell types or conditions, *UCP1* was not among the target genes of HIF1A or HIF1B (ChIP-Atlas), or there was no experimental evidence for HIF1A-HIF1B heterodimer binding to the *UCP1* promoter (TSS-5 KB) (ChIPSummitDB; EnhancerDB). Although, in several non-adipose cell lines, HIF1B (ARNT) binding to *UCP1* promoter was reported based on the ENCODE database. Notwithstanding, this HIF1A binding motif region is an accessible and active regulatory element of the *UCP1* promoter, as the histone methylation and acetylation state suggest (Additional file 10, Supplementary Fig. 5A; https://genome-euro.ucsc.edu; http://www.licpathway.net/ATACdb/) and other TFs and PoL2 bind to this DNA part (Fig. 4B; ChIPSummitDB, ENCODE database Additional file 10, Supplementary Fig. 5 A). The nucleotide sequence of the HIF1A-HIF1B RE and its immediate vicinity in humans also exists in the homologous promoter region of the paralog *UCP2* gene (Fig. 4C; EPD database; Additional file 10, Supplementary Fig. 5B), which is evolutionarily one of the closest descendants to *UCP1* [38]. In addition, both the ChIP-Atlas and the ChIPSummitDB database show, based on experimental data, that the HIF1A-HIF1B heterodimer does bind to this part of the *UCP2* gene promoter (Fig. 4D). According to the comparative genomic analysis of the UCSC genome browser, this is a phylogenetically new response element appearing only in primates and absent in the *UCP1* or *UCP2* promoter of non-primates mammals such as mice or dogs (Additional file 10, Supplementary Fig. 5 A,B,C) which makes the in vivo study difficult. Additionally, Fig. 4E shows the schematic representation of the *UCP1* promoter region (− 250--285) with the identified putative binding site of HIF1A-HIF1B (− 263) and many overlapping and proximal binding sites for other transcription factors enriched in this region as revealed using the EPD database (*p* < 0.0001). The emerging picture shows an extensive group of potential transcription factors that may alternate or co-interact to regulate transcriptional activation or repression of *UCP1* at this region and can directly modulate the thermogenic activity of the adipocytes according to actual needs and circumstances. HIF1A is a basic helix-loop-helix motif (bHLH) type TF, which contains two additional PAS domains and whose efficient DNA binding requires dimerization with another bHLH protein. Since the response element of HIF1A-HIF1B heterodimer (GRACGTGC; HIF1A response element: ACGTGC; JASPAR database http://jaspar.genereg.net/ [31];) is very similar to the canonical palindrome consensus sequence called E-box (CACGTG) of bHLH TFs, this opens up the possibility of fine-tuned regulation of *UCP1* gene expression by the Myc/Max/Mad network, Hey2/Hes1, TCFL5, NPAS2 and bHLHE40–41. In addition, ID proteins (Inhibitor of Differentiation 1–3), which appeared in the expanded white gene set, and show significantly increased expression in our *FTO* obesity-risk brown adipocyte samples, may be closely related to and involved in these regulatory processes by blocking the DNA binding and transactivation function of bHLH TFs [39] and thus hindering browning. An SNP identified in the HIF1A response element region (rs18006600) may also affect the binding probability of TFs and the regulation of *UCP1* gene expression (Fig. 4B; ChIPSummitDB). According to the expression profile of these overlapping TFs (Fig. 4F, Upper part), which have the potential to prevent HIF1A-HIF1B binding, most of them could repress the *UCP1* gene-expression, as they show higher expression in our *FTO* obesity-risk brown adipocyte samples, and only *bHLHE40* (*DEC1*) and *bHLHE41* appear as possible positive regulators according to their increased expression in our *FTO* normal genotype samples. The other transcriptional factors nearby to the HIF1A-HIF1B heterodimer could cooperate with it to form a complex and/or interact with other regulators and cofactors in the *UCP1* promoter. Their relative expression profile suggests that most of them also may suppress *UCP1* expression, as they are more expressed in *FTO* obesity-risk brown adipocyte samples, but RXRA-Nr1h3(LXRA) heterodimer and the znf740 could act as activators as their expression is higher in the *FTO* normal genotype samples (Fig. 4F; Lower part).

Regulation of HIF1A protein level in the cell is primarily mediated by inhibition of continuous proteasome degradation initiated by the enzyme Prolyl-hydroxylase: thus, gene expression data do not provide sufficient information on the level of HIF1A. Therefore, we examined its presence in differentiated human Adipose Stromal Fraction Cells (hASFCs) of abdominal subcutaneous origin (Abdominal-SC, Methods). Based on preliminary data in the subcutaneous derived hASFCs, HIF1A is not or very weakly stabilized in the in vitro differentiated adipocytes kept at 21% O_2_ level. However, the addition of a cAMP analog used for thermogenic activation (as demonstrated by UCP1 expression) elevated HIF1A stabilization, similar to hypoxic conditions (1% O_2_ level; Methods; Additional file 10, Supplementary Fig. 6). Consequently, the cell state-dependent presence of HIF1A via a broad range of regulated genes and highly dynamic DNA occupancy could influence the thermogenic phenotype formation through fine-tuned spatiotemporal gene expression regulation in matured adipocytes. This finding may indicate that the regulatory modules and elements revealed by network analysis can generate testable hypotheses.

## Discussion

Exploring a broad database can blur the variation resulting from specific experimental systems or the sample origin, thus highlights the common biological differences. In the case of BATLAS (large cohort studies [21];) and ProFAT (meta-analyzes of publicly available data-sets [22];), well usable marker-gene sets for determining the browning capacity of the fat cells or tissues were identified. Based on the similarity of the pathways defined by the marker-genes in the two gene sets, we thought it was worth generating an expanded protein and gene set for deeper analysis of thermogenesis regulation in adipocytes.

Analysis of the expanded gene set clearly shows that the direction of the regulatory processes of gene expression can be interpreted differently if comparative data from preadipocytes are also included. The differentiation process appears to induce the expression of a number of genes, which are highly expressed in the thermogenic phenotype, but also moderately appear in the less thermogenic samples. It means that these genes are not suppressed at the chromatin level even in obesity-risk genotype brown adipocytes, which can ensure the readiness of immediate heat production by proper stimulation. These genes may be positive modulators of the browning process; however, by itself, their expression in a white adipocyte may not be able to turn on thermogenesis. Several recent studies suggest that epigenome alterations can influence adipose tissue plasticity in mice and humans [40–45]. These studies collectively imply that it is primarily the repression of genes/pathways that potentially trigger the transcriptional reprogramming of adipocytes, which corroborates our conclusion from the relative expression profile of expanded gene lists when preadipocytes were also considered. Suppression of certain brown or white pathway genes (indirect regulation of thermogenesis by getting rid of interfering proteins) may result in more decisive physiological consequences in contrast to the differentially upregulating processes. For example, a recent study using single nucleus RNA sequencing was able to identify a rare population of cells in brown and white adipose tissues that express the enzyme that negatively regulates thermogenesis, suggesting that downregulation of ALDH1A1 is required for heat-producing adipocyte phenotype [12]. In summary, most of the well-known brown marker-genes that seem to be required for the thermogenic brown adipocyte state may be essential for proper differentiation too, although, at a moderate level, which supports the previously demonstrated phenomenon that the cafeteria diet induces adipocyte thermogenesis in addition to differentiation [46]. Their amount may affect browning capacity and promote hyperplasia, while a bit paradoxically, the thermogenic state may be induced or promoted through broad gene repression processes.

By using network analyses, we can explore the biological processes, signaling pathways, and proteins that may become important in thermogenesis from a different perspective. Growing evidence indicates that mice and adult human BAT (Brown Adipose Tissue) have high heterogeneity [8, 12, 47] and plasticity [48–50] as a response to external cues, which makes their characterization a challenge and may give contradictory results [10, 11]. Generally, the presence of proteins and their interaction in cells is highly dynamic, and with the expanded protein set, we can explore all the possible interactions of the protein network and identify the molecular signatures and pathways that potentially play a role in thermogenesis, which otherwise is conditional dependent. It could also lead to more generally applicable conclusions than single-state studies. In the analyses, the proteins belonging to the pathways that shape the brown and white phenotypes do not show one single integrated network but rather appeared in discrete clusters, which raises the possibility that different processes may form these phenotypes, and we need to know more about their transient states. Nevertheless, this network arrangement suggests that changing phenotype might be due to action from two directions: so that besides activating the browning processes, repression of molecular elements of the white phenotype may also be necessary to generate the desired thermogenesis without pathological consequences. By inducing or inhibiting the expression of proteins occupying a key position in the network or of a cluster, we may be able to initiate more diversified processes that result in the intended functional change. Among the identified central pathways and proteins, the role of the PI3K/PTEN/Akt/FoxO1 in the regulation of cellular energy metabolism and the thermogenesis of adipocytes has been highly investigated [51, 52], and it is well established that the proteasomal degradation of the PTEN protein leads to Akt activation and inhibited FoxO1-dependent *UCP1* expression in BAT [53]. Consistently, mice overexpressing PTEN are protected from metabolic damage: thus, we assume that PTEN positively regulates energy expenditure and brown adipose function [54].

The role of HIF1A, Androgen Receptor (AR), REL Proto-Oncogene, NFKB Subunit (REL), Runt Related Transcription Factor 1 (RUNX1), and TP53, which were spotted here, are less established in adipocyte thermogenesis. For example, studies have demonstrated the sex-specific effects of androgens on adipocyte function and suggest that androgens are essential for normal adipogenesis in males and can impair essential adipocyte functions in females; these sex hormones are factors that may explain, in part, the gender-dependent BAT thermogenic response [55, 56]. The role of inflammatory processes in adipose tissue remodelling is also of widespread interest, and the evolving assumption is that IKKβ/NFKB is activated by over-nutrition and weight gain in white adipose tissues leading to increased systemic and tissue inflammation and insulin resistance [57–59]. However, other studies reported that brown differentiation also induces the activation of NFKB and MAPK signaling pathways [60], and activation of these pathways resulted in improved insulin sensitivity; they conclude that NFKB promotes energy expenditure and inhibits adipose tissue growth [61]. These studies suggest that the functions of IKKβ/NFKB (REL) signaling in adipose tissue are very complex, and further studies are needed to untangle these versatile regulatory processes. Based on our network, NFKB1 belongs to the Linker genes, while NFKB2 and IKKβs belong to the white pathway genes. Similarly, TP53 seems to be a pleiotropic regulator; shaping adipocyte phenotype as depending on the adipose tissue depot, it could exert a positive (brown adipocytes) or negative (white adipocytes) effect on the specific adipogenic differentiation program: thus, it plays a role in maintaining the homeostatic state [62, 63], which is consistent with the fact that in our analysis, it belongs to the group of linker genes/proteins.

Each of the methods used to explore the molecular background of adipocyte phenotypes has some limitations. The recently used snRNA-seq approach attempts to estimate a different dimension of variability, which results from the heterogeneity of cells within tissues (in space and time) by obtaining the transcriptional profiles of the individual cells. However, these studies still suffer from variability caused by experimental conditions (e.g., the applied workflow, type of organism, in vitro vs in vivo experiment, ambient temperature, age, etc.). It has been recently reported [64] that systematic comparison of recovered cell types and their transcriptional profiles Iacross the workflows has protocol-specific biases. By comparing the results of recent snRNA-seq. studies, all of them explored large tissue heterogeneity, but different factors have been highlighted to be determinants in the formation of the thermogenic phenotype [13–15]. When we investigated the network position based-scores of these identified factors to estimate their role in adipose thermogenicity, we found good concordances. For example, in their single-nucleotide RNA-seq study, Biagi et al. [13] identified 7 transcription factors (PPARG, ERG1, STAT3, BHLHE40, ESR1, CEBPD, PPARD) whose expression positively correlated with UCP1 expression and five (PPARG, AR, ESR1, GATA2, Trp63) that correlated with low UCP1. When compared to our results, AR ranks first in the number of bridges list among the 3705 proteins (expanded protein list) and our core proteins determined by network analysis, indicating that it may have potentially outstanding significance in the linkage of the proteins that make up the network. Furthermore, it belongs to the group of genes/proteins of white phenotypic pathways, which also agrees with the result of Biagi, as AR was among the top 5 transcription factors in cells expressing low UCP1. PPARG has been described as dominant in both UCP1 high and low subpopulations; this is also consistent with our results, as PPARG belongs to the group of linker genes. Its position is also important, as it is the 28th of the 3705 proteins in betweenness centrality. ERG1 and BHLHE40 TFs appear in the *UCP1* promoter region among TFs that overlap/proximal with the HIF1A response element. When applying single-nucleus RNA-seq, Sárvári and colleagues [15] tried to uncover all major cell types in the epididymal adipose tissue (eWAT). They were able to separate three adipocyte subpopulations in epididymal adipose tissue, which show a change in obesity: lipogenic (LGA), lipid scavenging (LSA). and stressed lipid-scavenging adipocytes (SLSA). Many of the identified marker genes of these subpopulations appeared in our expanded gene-set and the protein network that we generated: LGA markers appear as brown pathway and linker genes, while the LSA and SLSA markers belong to the white pathways (except APOE, which is a linker gene). The same pattern is obtained when we looked for the identified obesity-regulated genes: those upregulated in lean mice belong to the brown and linker genes, while highly expressed genes in obese mice were found among the white pathway genes (except cd36, which is a linker gene). In another study [14], a metabolically-active mature adipocyte subtype was identified, which was characterized by robust expression of genes involved in thermogenesis and whose transcriptome was selectively responsive to IL10Ra deletion. Loss of this receptor in adipocytes promotes thermogenesis and confers obesity resistance. In our network, IL10 belongs to the white pathway and has a very high betweenness centrality score (position 85.). All of these support our proposition that the position of proteins in the interaction-network we generated can be a good predictor in estimating the role of a particular protein in the adipocyte thermogenic processes.

The role of HIF signaling in the regulation of adipocyte differentiation [65] and maintaining metabolic homeostasis under O_2_-deficient conditions has also been extensively investigated and explored [37, 66, 67]. In obese people, it has been shown that parts of the adipose tissue can become acutely or chronically hypoxic [68], which can contribute to the onset and progression of obesity-associated diseases [69, 70]. Besides this, many studies suggest that HIF1A/HIF2A/HIF3A may play a role in regulating adipocyte differentiation and thermogenesis in even a non-hypoxic environment [35, 71–74]; however, the molecular details have not been clarified. Tissue-specific HIF1A I.1 expression and protein accumulation have been reported at the early stage of adipogenesis in normoxic conditions [75], and it has also been demonstrated that norepinephrine without UCP1 expression can result in stabilized HIF1A in brown adipocytes [36]. However, the O_2_ concentration has not been studied at the cellular level; therefore, we cannot know whether the cells were actually in a state of normoxia or hypoxia. In addition, in a Genome-Wide Association Study that explored data of European-descent individuals, the *HIF1AN* rs17094222 loci was identified as a Body Mass Index (BMI) associated loci [76]: and in a recent large-scale epigenome-wide association study, it was found that DNA methylation at the *HIF3A* site was associated with BMI [77]. HIF1A was among the recruited proteins to the *UCP1 cis*-regulatory elements in response to the newly identified browning regulators, the FGF6/FGF9 stimulation in murine brown adipocytes [78]. Recently, Basse et al. showed that HIF1A expression is important for basal and beta-adrenergic stimulated (by isoproterenol) expression of glycolytic enzymes and necessary for maximum glucose metabolism in thermogenic adipocytes, reducing the intense mitochondrial function generated ROS and its damaging effect [37]. This is contrary to a study, which demonstrated that adipose HIF1A overexpression inhibits thermogenesis and cellular respiration in brown adipose tissue, promoting obesity in the setting of reduced ambient temperature [74]. Notwithstanding, although its role in shaping the thermogenic adipocyte phenotype remains to be clarified, the hitherto unexplored possibility that HIF1A directly binds to the promoter region and regulates *UCP1* gene-expression, may shed new light on the regulation of thermogenicity of adipocytes and provide promising targets for the pharmacological treatment of obesity.

## Conclusions

**Fig. 5:**
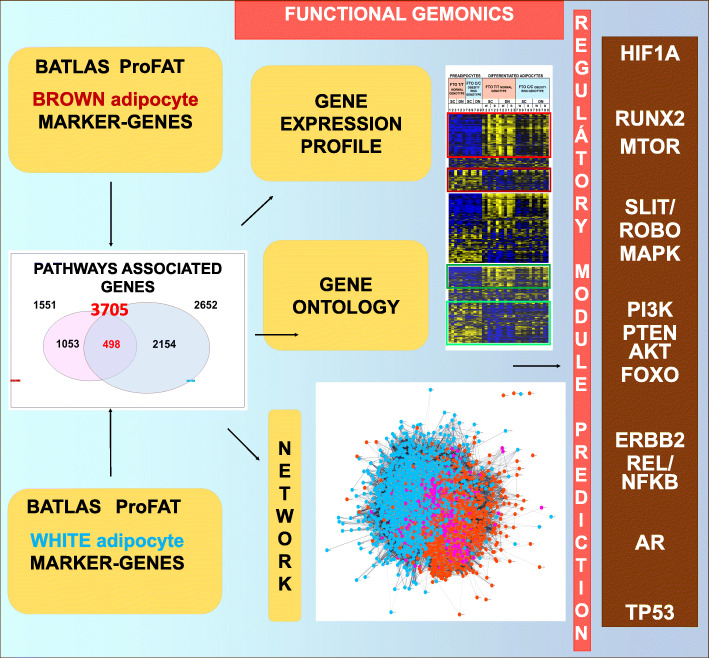
Graphical summary of the applied method for identifying the potential regulatory elements in adipocyte thermogenicity

Our new approach to exploring the molecular background of adipocyte phenotypes by integrating data from complex databases shows that entire pathways may better characterize brown and white adipocytes than marker-genes, allowing us to reveal the potentials of thermogenesis regulation in adipose tissues. It reduces the limitations of other approaches, such as differences in the experimental conditions of particular studies, and has the advantage of identifying markers that are not primarily regulated at the gene expression level (Fig. 5). The applied methodology has its limitations, including that only the regulatory elements manifesting at the protein level are covered, and the role of non-coding regions (e.g., miRNA, lncRNA), SNPs, metabolites cannot be studied directly. Our knowledge about the proteins of the characteristics pathways forming adipocyte phenotypes and their interaction system may also be incomplete. Therefore, we strive to continuously update the adipocyte database of the expanded gene/protein network. According to our analysis, different processes shape the brown and white adipocyte phenotypes, and the thermogenic phenotype may require simultaneous repression of whitening and induction of browning (Fig. 5). Locating the HIF1A response element in the *UCP1* promoter implies that HIF1A may play a role in regulating thermogenesis, also suggesting that new regulatory elements can be explored from the generated expanded database.

## Supplementary Information

**Additional file 1.** KEGG pathway analyses of the BATLAS (98 genes), ProFAT (53 genes) Brown marker-genes. Excel working sheets (.xlsx) show the annotated proteins for the enriched pathways based on KEGG database.

**Additional file 2.** REACTOME pathway analyses of the BATLAS (98 genes) and ProFAT (53 genes) Brown marker-genes. Excel working sheets (.xlsx) show the annotated proteins for the enriched pathways based on REACTOME database.

**Additional file 3.** KEGG pathway analyses of the BATLAS white marker-genes (21 genes). Excel working sheets (.xlsx) show the annotated proteins for the enriched pathways based on KEGG database.

**Additional file 4.** REACTOME pathway analyses of the BATLAS white marker-genes (21 genes). Excel working sheets (.xlsx) show the annotated proteins for the enriched pathways based on REACTOME database.

**Additional file 5.** List of the genes belongs to the downregulated gene cluster (dark red box; 175 genes in Fig. 2 B) identified by the hierarchical cluster analyses of the gene-expression data of the expanded Brown gene-set and their GSEA. Excel working sheets (.xlsx) show the annotated proteins for the enriched pathways based on KEGG and REACTOME database.

**Additional file 6: Supplementary Table 1.** List of BATLAS Marker-genes.

**Additional file 7: Supplementary Table 2.** List of ProFAT Marker-genes.

**Additional file 8: Supplementary Table 3.** Significantly enriched pathways based on BATLAS, ProFAT marker-genes, and genes differentially expressed in human neck adipocyte samples based on the presence of the FTO obesity-risk allele. (A) KEGG pathways (B) REACTOM pathways identified by BATLAS, ProFAT Brown marker-genes and the genes expressed lower in *FTO* C/C (obesity risk) samples. (C) KEGG pathways (D) REACTOM pathways identified by BATLAS White marker-genes and the genes expressed Higher in *FTO* C/C samples. Red letters: Common Pathways that enriched based on BATLAS and ProFAT marker-genes; Blue letters: Common Pathways in *FTO* based DEGs and BATLAS or ProFAT; Italic font: Common enriched Pathways in *FTO* based DEGs and BATLAS and ProFAT.

**Additional file 9:** **Supplementary Table 4.** Reactome and KEGG pathway analyses of the LINKER genes. The table shows the most significantly enriched Reactome and KEGG pathways of the LINKER genes (498), which supports the relevant composition of the expanded protein/gene set.

**Additional file 10:** **Supplementary Fig. 1.** Marker-genes of BATLAS and ProFAT. Venn diagrams show the number of marker-genes from BATLAS and ProFAT databases. **Supplementary Fig. 2.** Flowcharts of Data Collection and analyzes pipelines. (A) Main steps of the generation of the expanded protein/gene-set. (B) Exploration steps of the Expanded protein/gene set. (C) Investigation pipeline of the 30 core module proteins of the expanded protein-set network. **Supplementary Fig. 3.** Relative gene-expression profile of marker-genes from BATLAS and ProFAT. Relative gene-expression profile of marker-genes from BATLAS (A) and ProFAT (B) based on the presence of FTO obesity-risk alleles in differentiated Subcutaneous and Deep-neck adipocytes *n* = 6; samples1–3 with *FTO* T/T obesity-risk-free allele (donor 1–3) and samples 7–9 with C/C-obesity-risk alleles (donor 7–9) the *FTO* T/C heterozygous samples are not shown (donor 4–6) which was presented in some case in the paper Tóth et al., 2020. (BATLAS (C) and ProFAT (D) marker-genes expression profile based on the presence of FTO obesity-risk alleles in pre and differentiated Subcutaneous and Deep-neck adipocytes *n* = 6. **Supplementary Fig. 4.** Unsupervised MCL of the Interactome network of the 3705 Brown and White Pathways genes encoded proteins generated sub-clusters (Cytoscape); Nodes represent proteins; edges represent protein-protein interactions. Figure only show clusters contains more than 3 proteins. Cream colour highlights clusters contains 8 or more proteins. Red nodes: Brown pathway proteins; Blue nodes: White pathway proteins; Magenta: proteins appeared in both Brown and White Pathway (Linker proteins). **Supplementary Fig. 5.** Comparative genomic analysis by UCSC genome browser. (A) UCSC genome browser overview of the *UCP1* promoter where HIF1A binding sequence is enriched (red box). It is show the histone methylation state, the Pol2 binding activity, the experimentally supported binding TFs and alignments of orthologues sequence from other species (highlighted in green); Strand [+] (https://genome-euro.ucsc.edu/; EPD Viewer HUB). (B) UCSC genome browser overview of the *UCP2* promoter where HIF1A binding sequence is enriched (red box). It is show the histone methylation state, the Pol2 binding activity, the experimentally supported binding TFs and alignments of orthologues sequence from other species (highlighted in green); Strand [+] (https://genome-euro.ucsc.edu/; EPD Viewer HUB). (C) Multiz Alignments of 100 Vertebrates data show the detailed sequence homology of the different species in the *UCP1* promoter where HIF1A binding sequence is enriched (red box) (Further species with no homologous sequence did not shown (https://genome-euro.ucsc.edu/cgi-bin/hgc?c=chr4&l=140569209&r=140569243&o=140569209&t=140569243&g=multiz100way&i=multiz100way&db=hg38). **Supplement Fig. 6.** Effect of thermogenic induction and Hypoxic condition on the protein level of HIF1A and UCP1. Immunoblots show HIF1A and UCP1 protein levels in cAMP analog-induced thermogenesis (16 h) and hypoxic environment (16 h) in differentiated adipocyte sample derived from human subcutaneous stromal vascular fraction, using beta tubulin (TUB) as a loading control. SC: Subcutaneous.

## Data Availability

The datasets of RNA-seq data from human studies reported in this paper supporting the conclusions of this article are available in the [Sequence Read Archive (SRA) database] repository, under accession number PRJNA607438 [https://www.ncbi.nlm.nih.gov/sra]. Algorithms for calculation of the Betweenness Centrality Score and Number of Bridges based on Protein Network Interaction confidence values identified by STRING computational tool was developed in this work in R programming environment. Project name: brownRNA; Project home page: https://github.com/zbartab/brownRNA; Operating system(s) Platform independent, Programming language: R, Other requirements: Igraph R package, License: public domain, Any restrictions to use by non-academics: none; We have also created an online platform, named AdipoNET, for the convenient use of the protein interaction-network position based data (betweenness centrality and number of bridges scores) in practice, which is freely available through browser. This online tool can help to predict the potential role of a given protein/gene in shaping adipocyte phenotype. Project name: adiponet; Project home page: https//adiponet.com, Operating system(s): Platform independent, Programming language: PHP, Other requirements: none, License: Creative Commons CT-4.0, Any restrictions to use by non-academics: none; All basic, supplementary and additonal data are deposited at the Mendeley Database (https://data.mendeley.com/datasets/hsj8wsfz7t/draft?a=fe332df2-48c7-42ed-b6aa-b635da0c6cac).

## Abbreviatons

ADCY4: Adenylate cyclase 4
ADP: Adenosine diphosphate.
ADRA2C: Adrenoceptor alpha 2C
AKT1: v-akt murine thymoma viral oncogene homolog 1
ALDH18A1: Aldehyde dehydrogenase 18 family, member A1
ALDH1A1: Aldehyde dehydrogenase 1 family, member A1
APOE: Apolipoprotein E
AR: Androgen receptor
ARID5B: AT-rich interactive domain-containing protein 5B
ARNT: Aryl hydrocarbon receptor nuclear translocator
ATP: Adenosine triphosphate
B: Brown
BAT: Brown Adipose Tissue
BDNF: Brain-derived neurotrophic factor
bHLH: Basic helix-loop-helix motif
bHLHE40-41: Basic helix-loop-helix family, member e40-41
BMI: Body Mass Index
C: Cytosine
cAMP: Cyclic adenosine monophosphate
CD36: CD36 molecule (thrombospondin receptor)
CEBPD: CCAAT/enhancer binding protein (C/EBP), delta
Chip: Chromatin immuno-precipitation
CKMT1A: Creatine kinase, mitochondrial 1A
CO2: Carbon-dioxide
COMP: Cartilage oligomeric matrix protein
CPT1A: Carnitine palmitoyltransferase 1A (liver)
CPT1B: Carnitine palmitoyltransferase 1B (muscle)
CSF2RA: Colony stimulating factor 2 receptor, alpha, low-affinity (granulocyte-macrophage)
DECR1: 2,4-dienoyl CoA reductase 1, mitochondrial
DHRS9: Dehydrogenase/reductase (SDR family) member 9
DIO2: Deiodinase, iodothyronine, type II
DN: Deep neck
DNA: Deoxyribonucleic acid
DUSP6: Dual specificity phosphatase 6
EGR1: Early growth response 1
ELOVL3: ELOVL fatty acid elongase 3
EPD: Eukaryotic Promoter Database
ERBB2: v-erb-b2 avian erythroblastic leukemia viral oncogene homolog 2
ESR1: Estrogen receptor 1
eWAT: Epididimal white adipose tissue
FABP3-4: Fatty acid binding protein 3, muscle and heart (mammary-derived growth inhibitor)
FDR: Fals discovery rate
FGF1: Fibroblast growth factor 1 (acidic)
FN1: Fibronectin 1
FoxO1: Forkhead box O1
FTO: Fat mass and obesity-associated
GAPDH: Glyceraldehyde-3-phosphate dehydrogenase
GATA2: GATA binding protein 2
GNAO1: Guanine nucleotide binding protein (G protein), alpha activating activity polypeptide O
GNB3: Guanine nucleotide binding protein (G protein), beta polypeptide 3
GNG7: Guanine nucleotide binding protein (G protein), gamma 7
GPX3: Glutathione peroxidase 3 (plasma)
GPX8: Glutathione peroxidase 8 (putative)
GSEA: Gene Set Enrichment Analyses
HACD4: 3-Hydroxyacyl-CoA Dehydratase 4
HAPLN1: Hyaluronan and proteoglycan link protein 1
hASFCs: Human Adipose Stromal Fraction Cells
Hes1: Hairy and enhancer of split 1, (Drosophila)
Hey2: Hairy/enhancer-of-split related with YRPW motif 2
HIF1A: Hypoxia Inducible Protein 1
HIF1AN: Hypoxia inducible factor 1, alpha subunit inhibitor (FIH1)
HIF1B: Hypoxia Inducible Protein Beta
HK2: Hexokinase 2
HRAS: Harvey rat sarcoma viral oncogene homolog [Source:HGNC Symbol;Acc:5173]
ID1: Inhibitor of DNA binding 1, dominant negative helix-loop-helix protein
IGF2: Insulin-like growth factor 2 (somatomedin A)
IKKβ: Inhibitor of nuclear factor kappa-B kinase subunit beta
IL11: Interleukin 11
IL6: Interleukin 6 (interferon, beta 2) [Source:HGNC Symbol;Acc:6018]
INS: Insulin
INSR: Insulin receptor
IRX3,5: Iroquois-class homeodomain protein 3
KEGG: Kyoto Encyclopedia of Genes and Genomes
KO: Knock out
LDHB: Lactate dehydrogenase B
LEFTY2: Left-right determination factor 2
LHPP: Phospholysine phosphohistidine inorganic pyrophosphate phosphatase
LIPE: Lipase, hormone-sensitive
lngRNA: long non coding RNA
LPAR3: Lysophosphatidic acid receptor 3
LPIN1: Lipin 1
LPL: Lipoprotein lipase
LTC4S: Leukotriene C4 synthase
LXRA: Liver X receptor alpha
Mad: Mothers against dpp
MAPK: Mitogen-activated protein kinase
MAPK3: Mitogen-activated protein kinase 3
Max: MYC associated factor X
MCL: Markov Clustering
MGST2: Microsomal glutathione S-transferase 2
miRNA: micro RNA
mRNA: messenger RNA
mTOR: mechanistic target of rapamycin (serine/threonine kinase)
MYC: v-myc avian myelocytomatosis viral oncogene homolog
N2: Nitrogen
NAFLD: Non-alcoholic fatty liver disease
NFKB: Nuklear faktor kappa B
NNMT: Nicotinamide N-methyltransferase
NOX4: NADPH oxidase 4
NPAS2: Neuronal PAS domain protein 2
Nr1h3: Nuclear receptor subfamily 1, group H, member 3
O2: Oxygen
PARK7: Parkinson protein 7
PAS: Per-Arnt-Sim
PC: Pyruvate carboxylase
PDK1-4: Pyruvate dehydrogenase kinase, isozyme 1
PGC1: Peroxisome proliferator-activated receptor gamma, coactivator 1 alpha
PI3K: Phosphoinositide 3-kinase
PIP3: Phosphatidylinositol (3,4,5)-trisphosphate
PLIN1-5: Perilipin 1
PPARD: Peroxisome proliferator-activated receptor delta
PPARG: Peroxisome proliferator-activated receptor gamma
PRDM16: PR domain containing 16
PTEN: Phosphatase and tensin homolog
RE: Response Element
REL: v-rel avian reticuloendotheliosis viral oncogene homolog
RNA: Ribonucleic acid
ROBO: Roundabout homolog
ROS: Reactive Oxygen Species
RPL3: Ribosomal protein L3
RPS2: Ribosomal protein S2
RUNX2: Runt-related transcription factor 2
RXRA: Retinoid X receptor, alpha
SC: Subcutaneous
SCD5: Stearoyl-CoA desaturase 5
SLC2A4: Solute carrier family 2 (facilitated glucose transporter), member 4
SLIT: Slit Guidance Ligand
SNP: Single nucleotide polymorphism
snRNA-seq.: single-nucleus RNA sequencing
SOD2: Superoxide dismutase 2, mitochondrial
SRC: v-src avian sarcoma (Schmidt-Ruppin A-2) viral oncogene homolog
STAT3: Signal transducer and activator of transcription 3 (acute-phase response factor)
SUCLG1: Succinate-CoA ligase, alpha subunit
T: Thymine
TCA: Tricarboxylic acid cycle
TCFL5: Transcription factor-like 5 (basic helix-loop-helix)
TF: Transcription Factor
TGFA: Transforming growth factor, alpha
TGFB1: Transforming growth factor, beta 1
TP53: Tumour protein 53
Trp63: Transformation-related protein 63
TSS: Transcription Start site
UCP1-2: Uncoupling Protein 1
UCSC: University of California Genomics Institute
VCAM1: Vascular cell adhesion molecule 1
W: White
znf740: zinc finger protein 740
