# Supplementary material for: Regulatory modules of human thermogenic adipocytes: functional genomics of large cohort and Meta-analysis derived marker-genes
Source: BMC Genomics. 2021 Dec 11;22:886. doi: 10.1186/s12864-021-08126-8 (PMC8665548; doi:10.1186/s12864-021-08126-8)
Supplement: Supplementary file 9 — Additional file 9: Supplementary Table 4. Reactome and KEGG pathway analyses of the LINKER genes. The table shows the most significantly enriched Reactome and KEGG pathways of the LINKER genes (498), which supports the relevant composition of the expanded protein/gene set. [file 12864_2021_8126_MOESM9_ESM.docx]

| **LINKER PROTEINS ENRICHED REACTOME PATHWAYS** | **FDR** |
| --- | --- |
| Signaling by ERBB4 | 2.35e-55 |
| PIP3 activates AKT signaling | 2.28e-27 |
| RORA activates gene expression | 1.98e-20 |
| Unfolded Protein Response (UPR) | 1.79e-16 |
| Neurotransmitter receptors and postsynaptic signal transmission | 8.55e-12 |
| PI3K Cascade | 2.06e-08 |
| Opioid Signalling | 8.48e-08 |
| Apoptosis | 2.08e-07 |
| Interleukin-6 signaling | 7.06e-07 |
| Developmental Biology | 0.00014 |
| ROS, RNS production in phagocytes | 0.00025 |
| The citric acid (TCA) cycle and respiratory electron transport | 0.0018 |
| Hemostasis | 0.0029 |
| Formation of RNA Pol II elongation complex | 0.0035 |
| Metabolism | 0.0095 |
| Presynaptic depolarization and calcium channel opening | 0.0112 |
|  |  |
|  |  |
| **LINKER PROTEINS ENRICHED KEGG PATHWAYS** | **FDR** |
| Non-alcoholic fatty liver disease (NAFLD) | 1.14e-153 |
| **Thermogenesis** | **7.66e-150** |
| Huntington's disease | 7.20e-132 |
| Alzheimer's disease | 1.33e-129 |
| Retrograde endocannabinoid signaling | 2.57e-107 |
| Oxidative phosphorylation | 1.96e-87 |
| Parkinson's disease | 1.97e-86 |
| Relaxin signaling pathway | 7.47e-70 |
| Pathways in cancer | 1.14e-68 |
| Glucagon signaling pathway | 5.26e-66 |
| Insulin resistance | 8.14e-66 |
| Dopaminergic synapse | 1.96e-65 |
| Metabolic pathways | 1.22e-64 |
| Apelin signaling pathway | 1.42e-64 |
| Cholinergic synapse | 7.35e-64 |
| Insulin signaling pathway | 5.17e-62 |
| Longevity regulating pathway | 3.37e-61 |
| Kaposi's sarcoma-associated herpesvirus infection | 3.39e-60 |
| cGMP-PKG signaling pathway | 3.62e-60 |
| AMPK signaling pathway | 2.56e-57 |
| Circadian entrainment | 2.22e-56 |
| Adrenergic signaling in cardiomyocytes | 5.64e-53 |
| Glutamatergic synapse | 9.38e-53 |
| cAMP signaling pathway | 5.53e-52 |

**Supplementary table 4. Reactome and KEGG pathway analyses of the LINKER genes.**

The table shows the most significantly enriched Reactome and KEGG pathways of the LINKER genes (498), which supports the relevant composition of the expanded protein/gene set.
