# Supplementary material for: Regulatory modules of human thermogenic adipocytes: functional genomics of large cohort and Meta-analysis derived marker-genes
Source: BMC Genomics. 2021 Dec 11;22:886. doi: 10.1186/s12864-021-08126-8 (PMC8665548; doi:10.1186/s12864-021-08126-8)
Supplement: Supplementary file 10 — Additional file 10: Supplementary Fig. 1. Marker-genes of BATLAS and ProFAT. Venn diagrams show the number of marker-genes from BATLAS and ProFAT databases. Supplementary Fig. 2. Flowcharts of Data Collection and analyzes pipelines. (A) Main steps of the generation of the expanded protein/gene-set. (B) Exploration steps of the Expanded protein/gene set. (C) Investigation pipeline of the 30 core module proteins of the expanded protein-set network. Supplementary Fig. 3. Relative gene-expression profile of marker-genes from BATLAS and ProFAT. Relative gene-expression profile of marker-genes from BATLAS (A) and ProFAT (B) based on the presence of FTO obesity-risk alleles in differentiated Subcutaneous and Deep-neck adipocytes n = 6; samples1–3 with FTO T/T obesity-risk-free allele (donor 1–3) and samples 7–9 with C/C-obesity-risk alleles (donor 7–9) the FTO T/C heterozygous samples are not shown (donor 4–6) which was presented in some case in the paper Tóth et al., 2020. (BATLAS (C) and ProFAT (D) marker-genes expression profile based on the presence of FTO obesity-risk alleles in pre and differentiated Subcutaneous and Deep-neck adipocytes n = 6. Supplementary Fig. 4. Unsupervised MCL of the Interactome network of the 3705 Brown and White Pathways genes encoded proteins generated sub-clusters (Cytoscape); Nodes represent proteins; edges represent protein-protein interactions. Figure only show clusters contains more than 3 proteins. Cream colour highlights clusters contains 8 or more proteins. Red nodes: Brown pathway proteins; Blue nodes: White pathway proteins; Magenta: proteins appeared in both Brown and White Pathway (Linker proteins). Supplementary Fig. 5. Comparative genomic analysis by UCSC genome browser. (A) UCSC genome browser overview of the UCP1 promoter where HIF1A binding sequence is enriched (red box). It is show the histone methylation state, the Pol2 binding activity, the experimentally supported binding TFs and alignments of orthologues sequence from o [file 12864_2021_8126_MOESM10_ESM.pptx]

## Slide 1
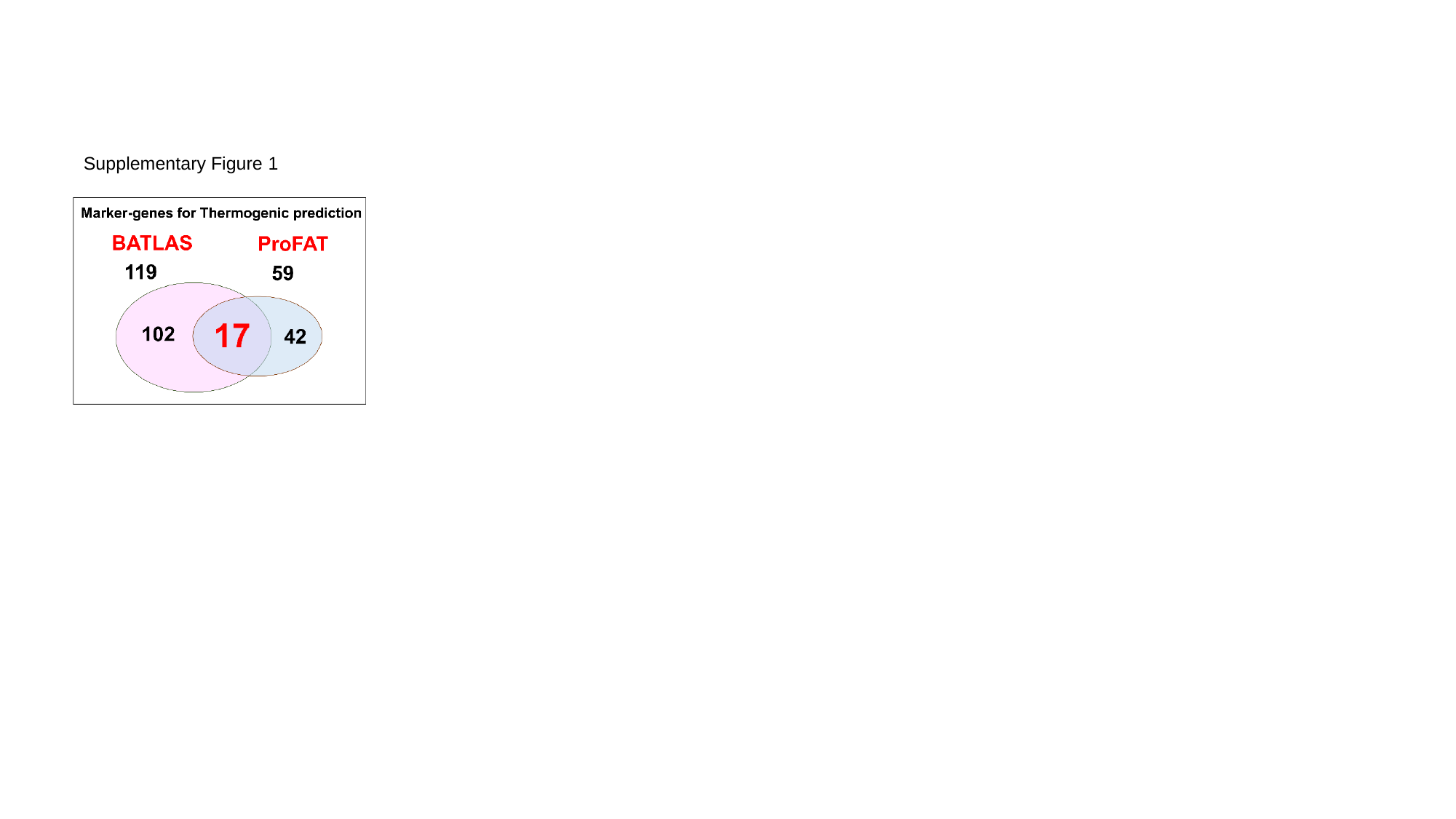

Supplementary Figure 1

## Slide 2
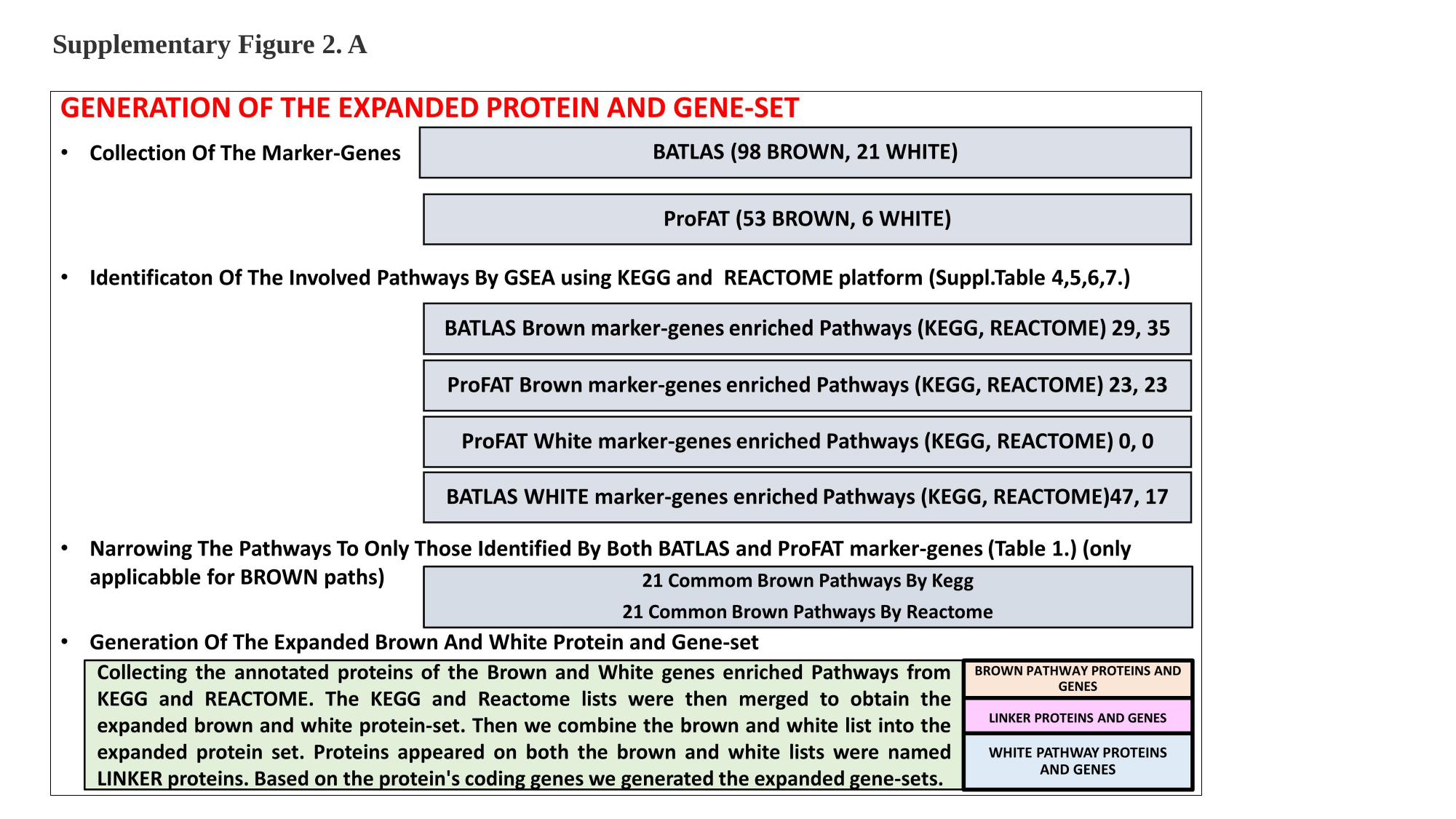

Supplementary Figure 2. A

## Slide 3
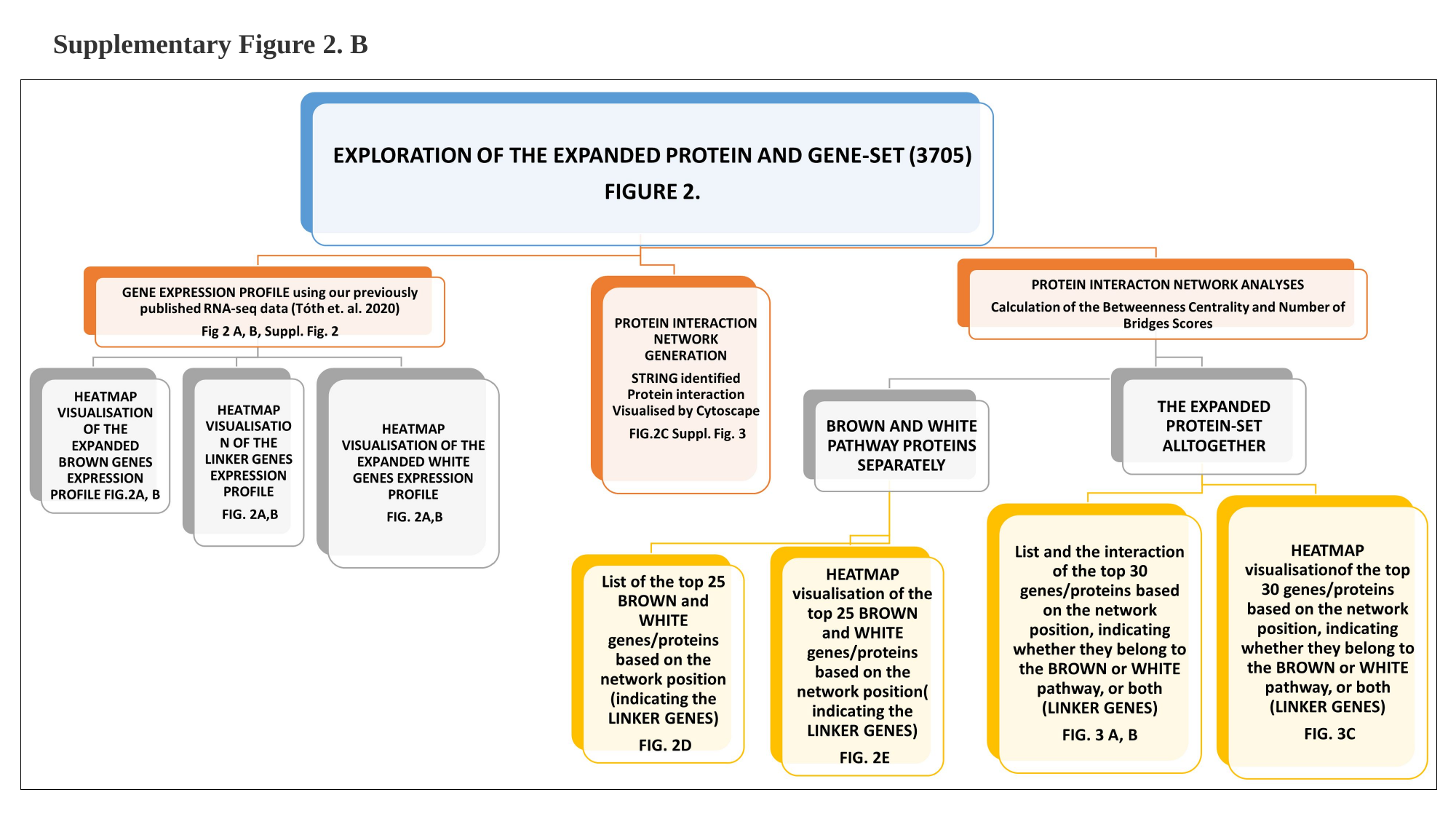

Supplementary Figure 2. B

## Slide 4
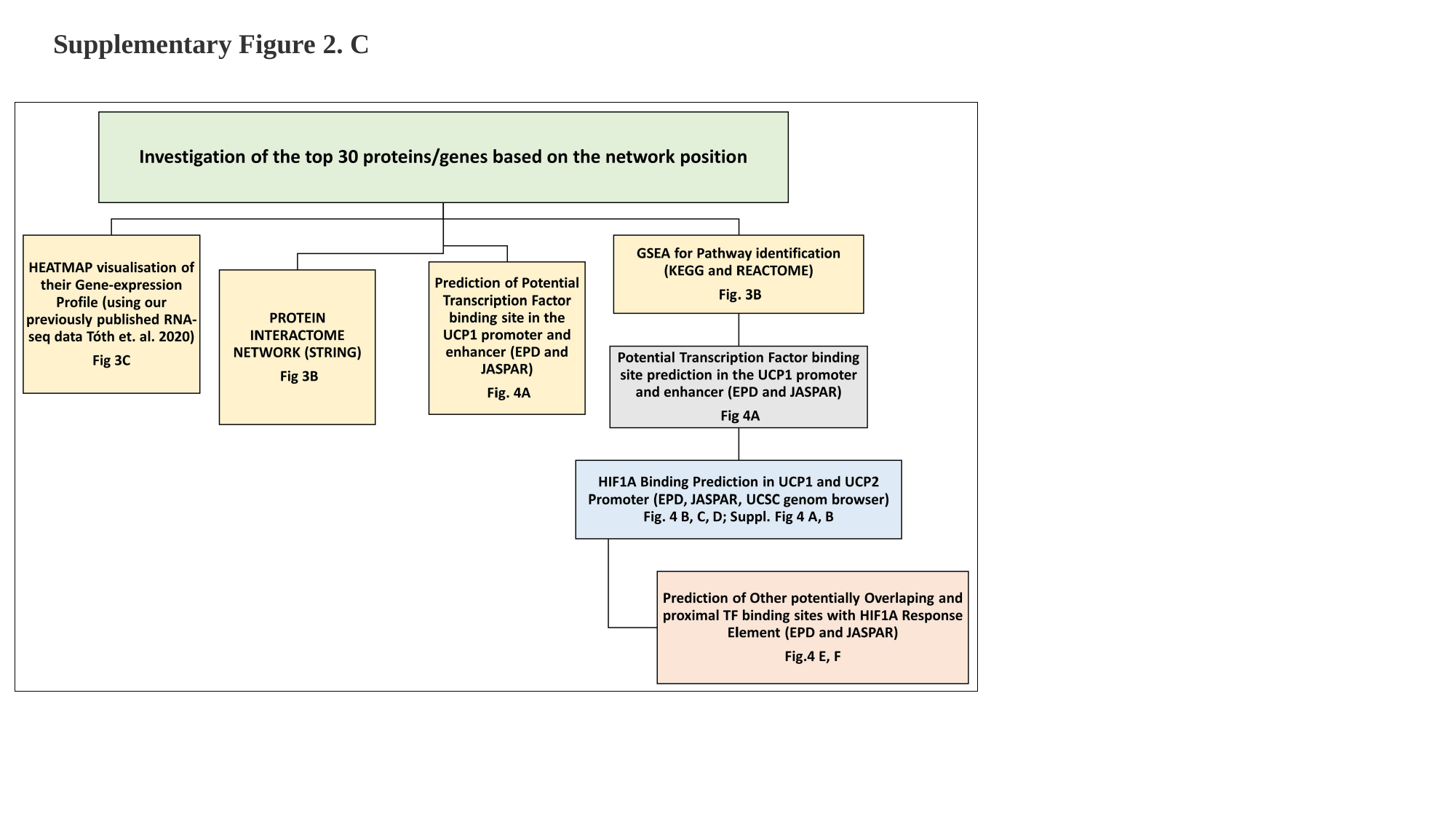

Supplementary Figure 2. C

## Slide 5
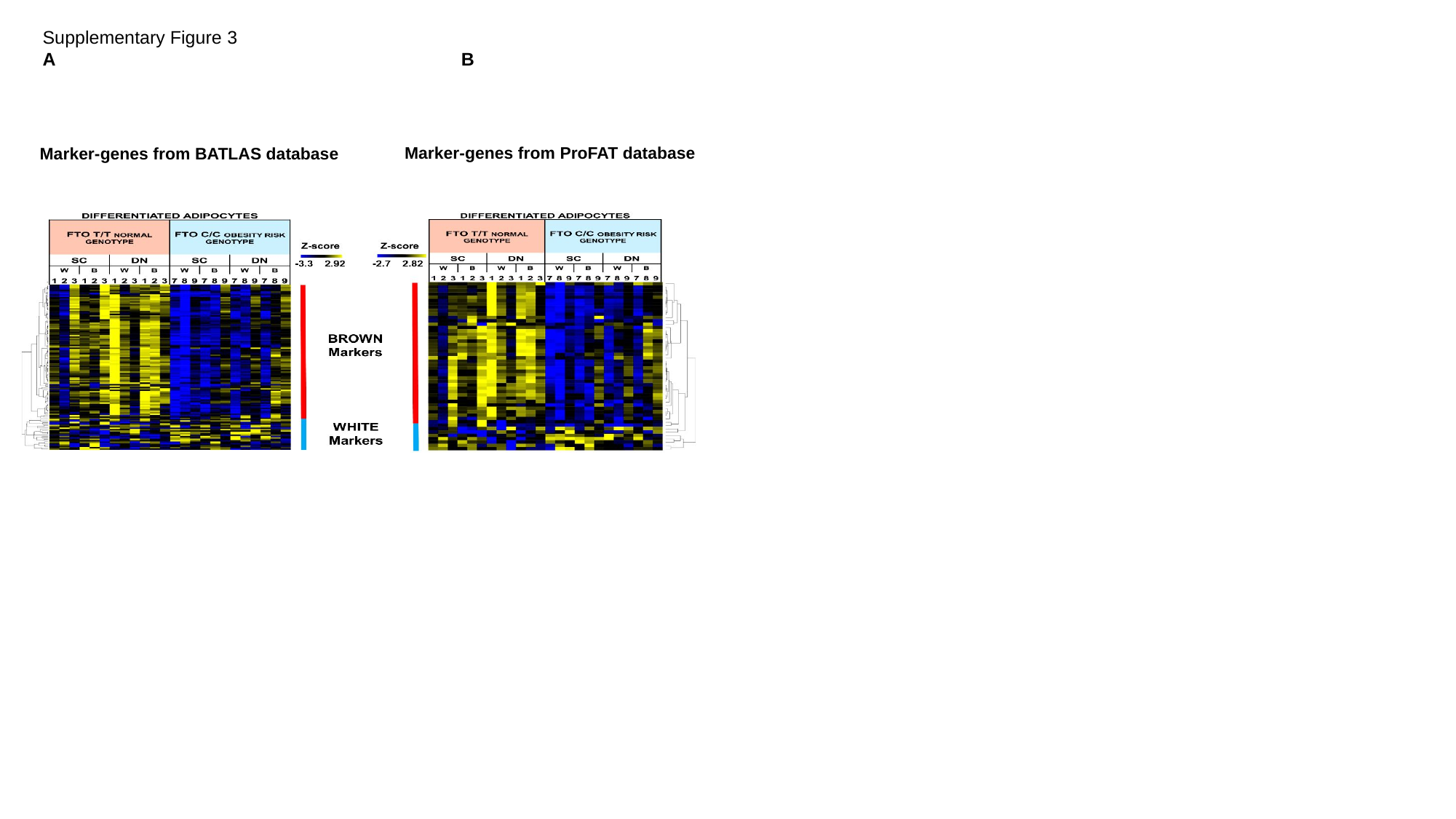

Supplementary Figure 3
A B
Marker-genes from BATLAS database
Marker-genes from ProFAT database

## Slide 6
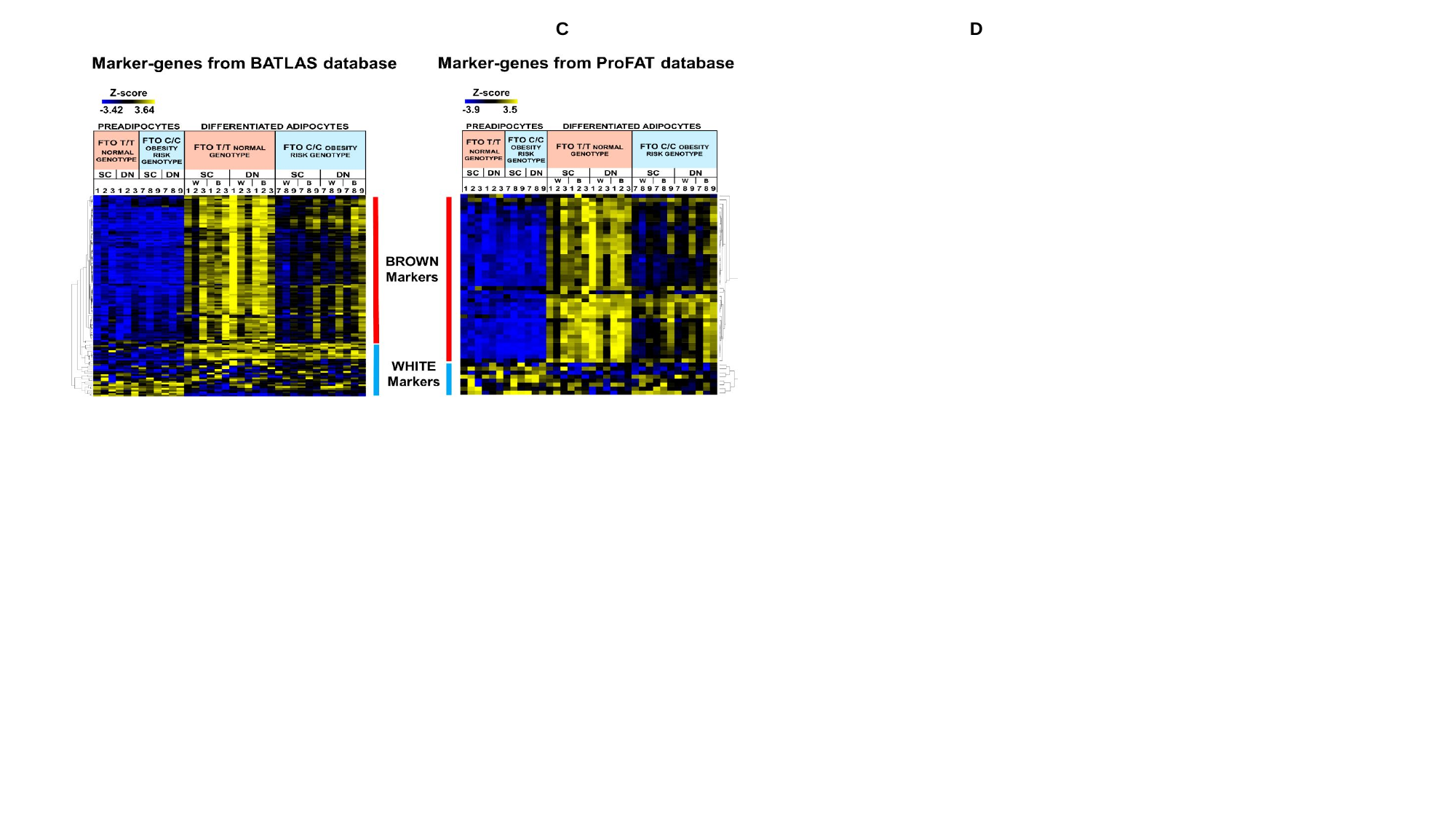

C D

## Slide 7
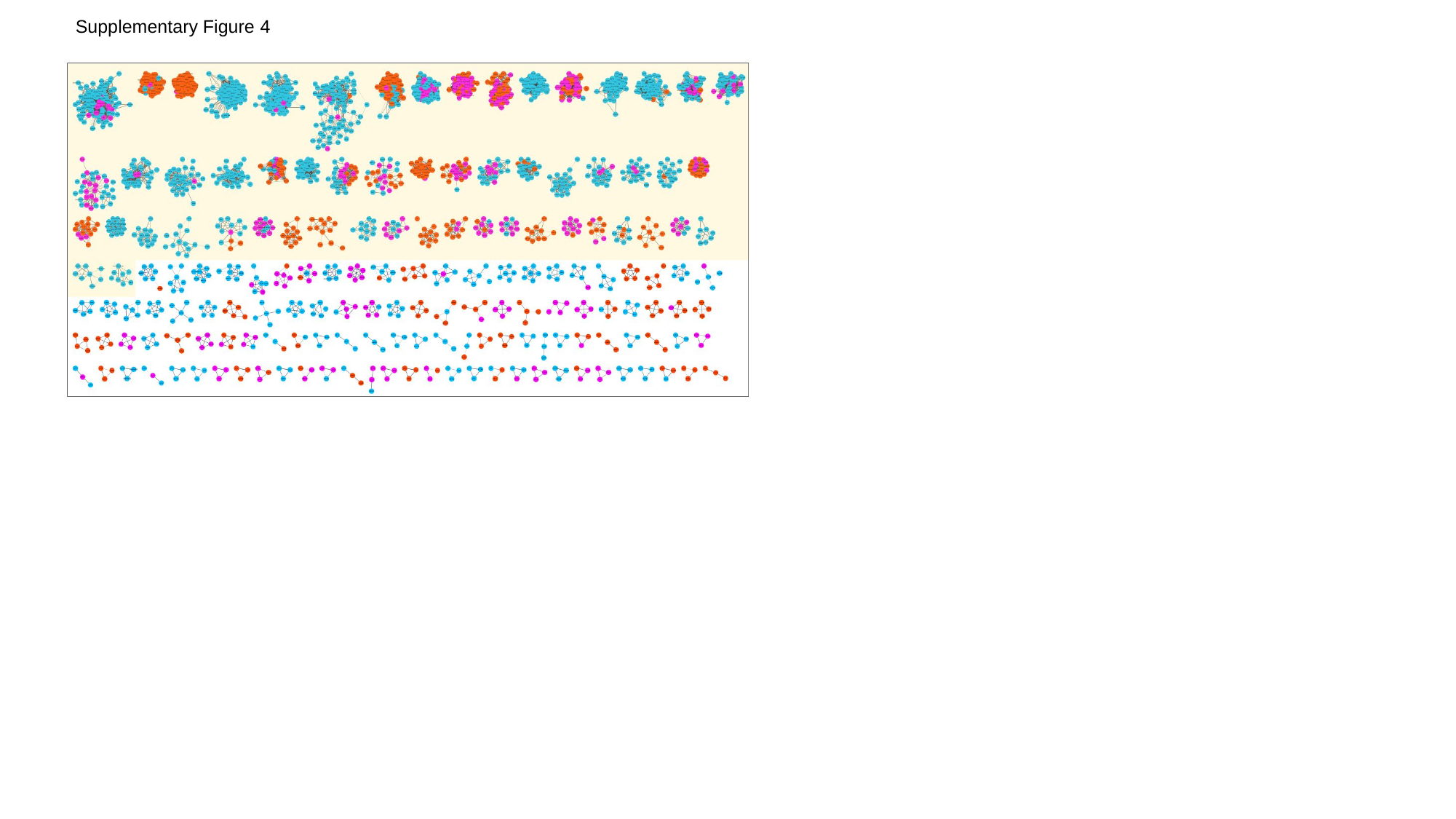

Supplementary Figure 4

## Slide 8
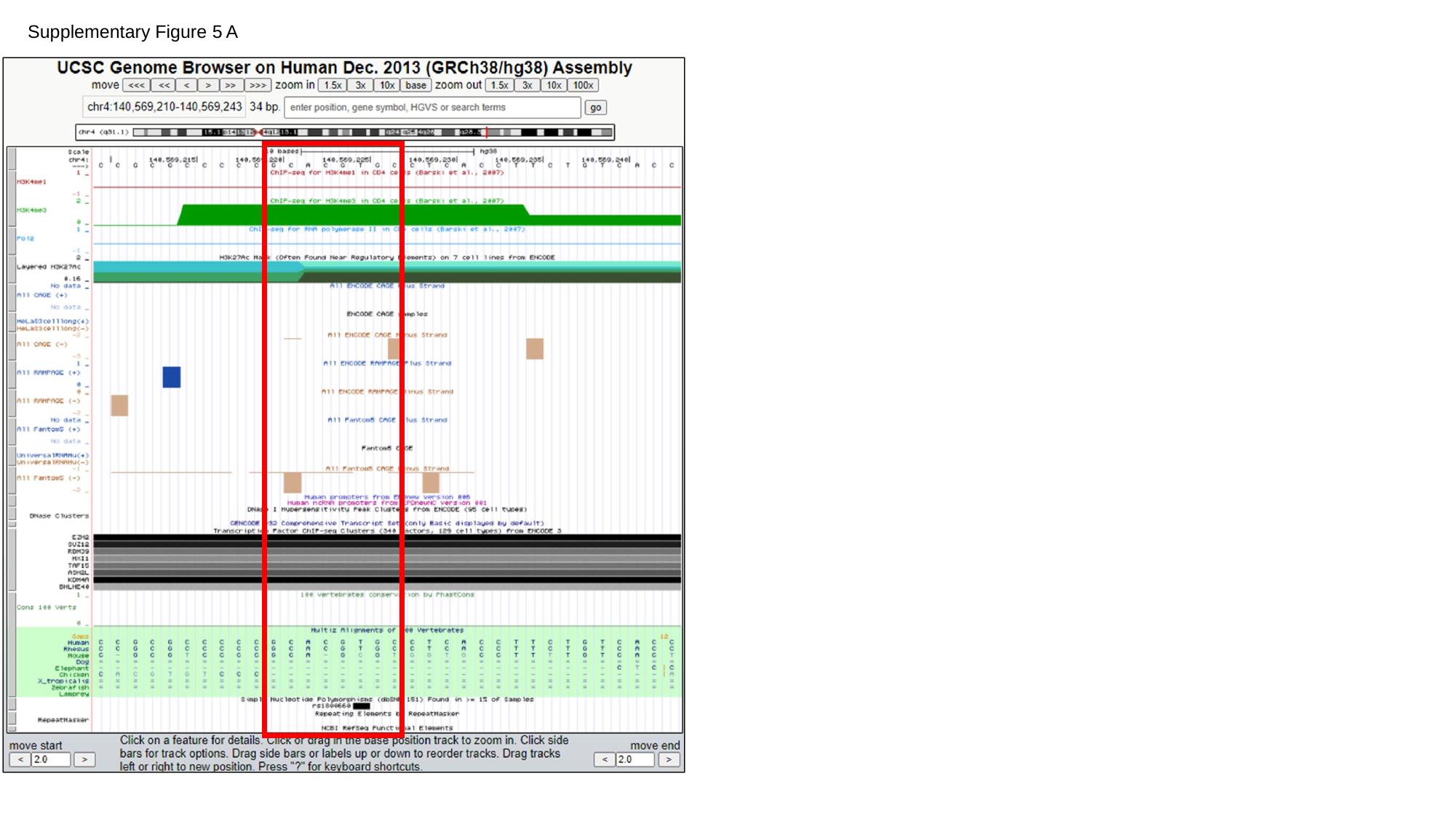

Supplementary Figure 5 A

## Slide 9
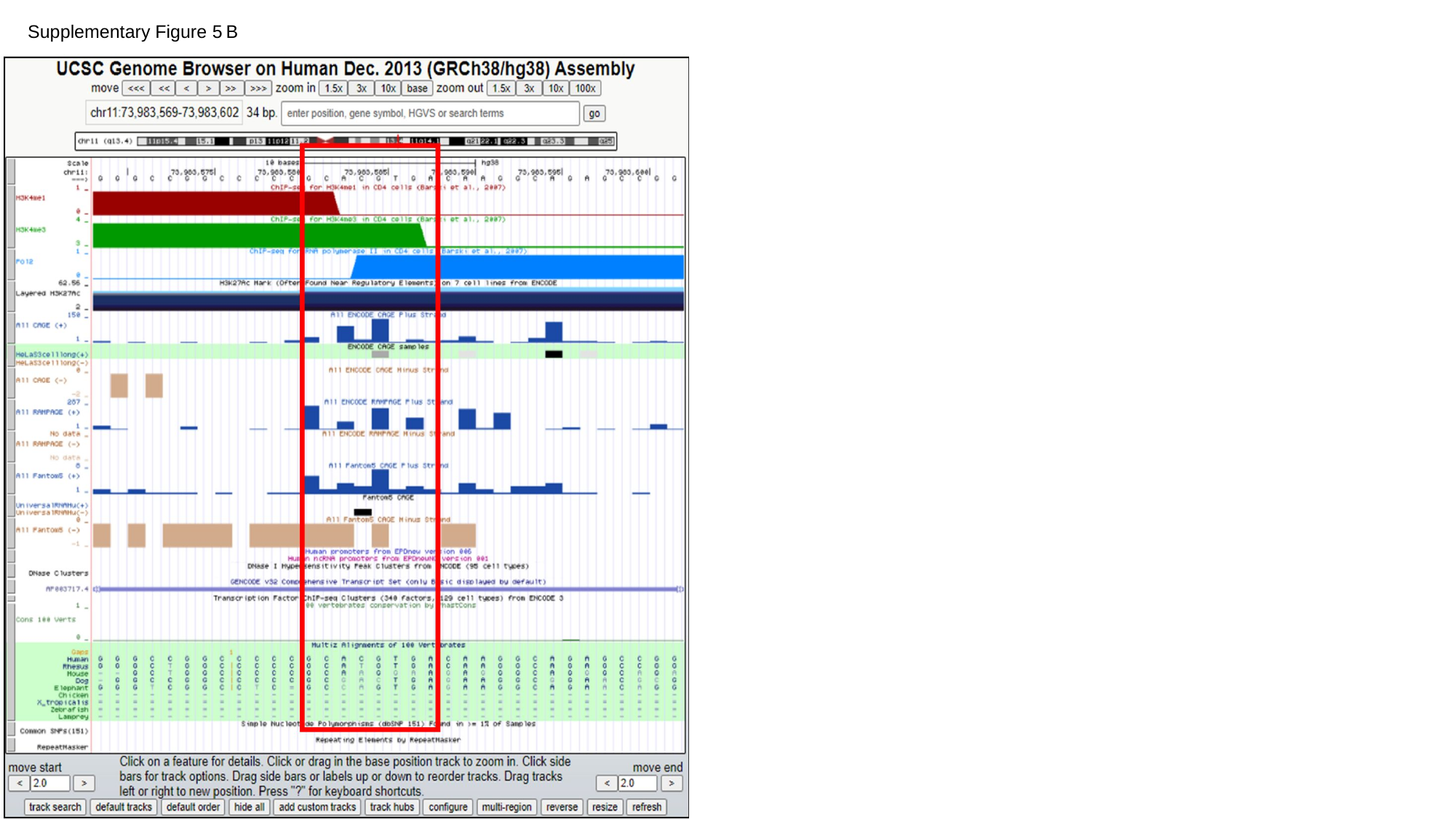

Supplementary Figure 5 B

## Slide 10
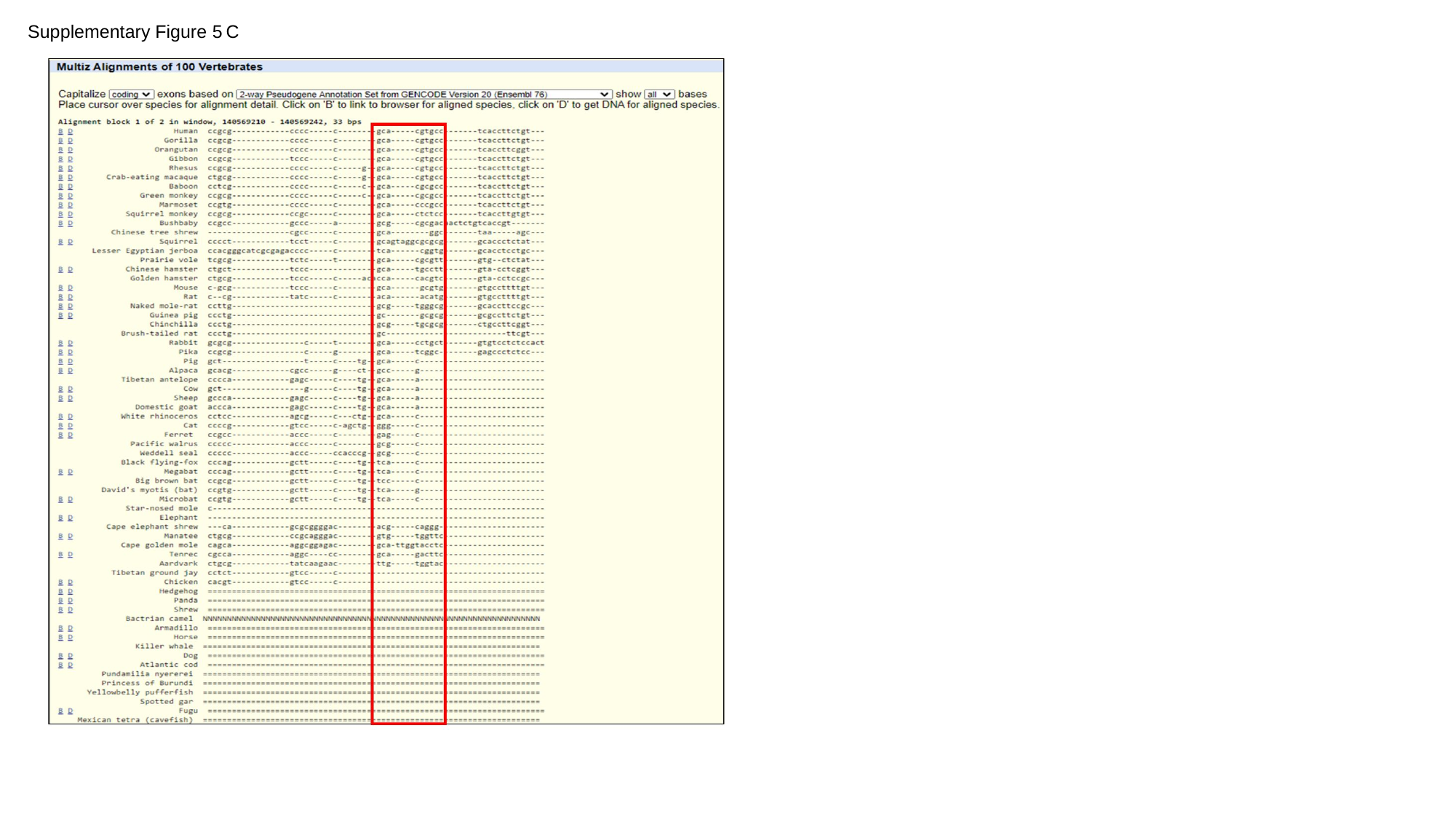

Supplementary Figure 5 C

## Slide 11
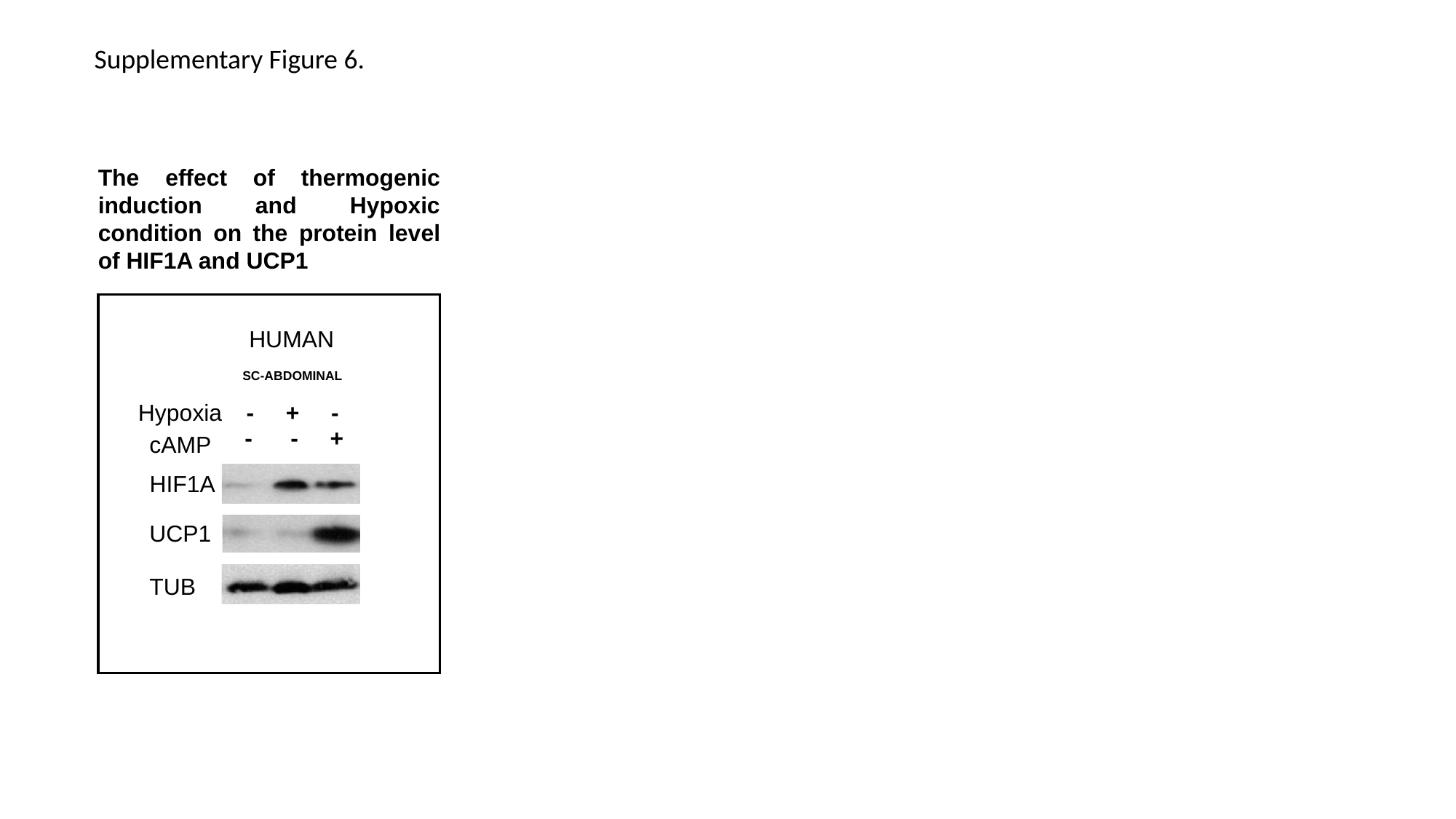

Supplementary Figure 6.
The effect of thermogenic induction and Hypoxic condition on the protein level of HIF1A and UCP1
HUMAN
 SC-ABDOMINAL
Hypoxia
cAMP
HIF1A
UCP1
TUB
- + -
- - +
